# Aspartate potentiates tobramycin against multidrug-resistant *Edwardsiella tarda* through enhancing proton motive force and membrane permeability

**DOI:** 10.1128/msystems.00794-25

**Published:** 2025-08-28

**Authors:** Ying-yue Zeng, Hui-yin Lin, Si-chen Yuan, Xuan-xian Peng, Hui Li

**Affiliations:** 1 State Key Laboratory of Bio-Control, School of Life Sciences, Southern Marine Science and Engineering Guangdong Laboratory (Zhuhai), Sun Yat-sen University 26469 , Guangzhou, China; 2 Laboratory for Marine Fisheries Science and Food Production Processes, Qingdao National Laboratory for Marine Science and Technology 474988 , Qingdao, China; University of Technology Sydney, Sydney, NSW, Australia

**Keywords:** aspartate, aminoglycosides, multidrug-resistant *Edwardsiella tarda*, proton motive force, membrane permeability

## Abstract

The antibiotic susceptibility/resistance of bacteria is influenced by their metabolic states. In tobramycin-resistant *Edwardsiella tarda* (LTB4-R_TOB_), a dynamic metabolic state is observed, characterized by a general reduction in most metabolites as the minimum inhibitory concentration increases. Among these, aspartate downregulation emerges as the most critical biomarker. Exogenous aspartate enhances the efficacy of tobramycin, enabling it to effectively kill both lab-evolved and clinically isolated multidrug-resistant (MDR) *E. tarda*, as demonstrated in an animal model. Aspartate increases intracellular tobramycin levels in a concentration- and time-dependent manner by promoting drug uptake. Metabolic reprogramming analysis of LTB4-R_TOB_ and clinically isolated MDR strain WY28 reveals that exogenous aspartate restores downregulated metabolites, primarily enhancing alanine, aspartate, and glutamate metabolism; the pyruvate cycle; and glycine, serine, and threonine metabolism. These changes elevate the proton motive force and membrane permeability, thereby increasing tobramycin uptake. The resulting intracellular drug concentration surpasses the bactericidal threshold, effectively overcoming resistance in both LTB4-R_TOB_ and clinical MDR *E. tarda*. These findings identify aspartate as a potent metabolic reprogramming agent that potentiates aminoglycosides by boosting intracellular drug accumulation, offering a strategy to combat antibiotic-resistant *E. tarda*.

**IMPORTANCE:**

*Edwardsiella tarda* is a significant pathogen that causes edwardsiellosis in aquaculture, resulting in substantial economic losses while also posing a global public health threat. The increasing prevalence of antibiotic-resistant *E. tarda* strains has further exacerbated this challenge. The present study used a metabolic state-reprogramming approach to identify an ideal biomarker as an antibiotic adjuvant to increase conventional antibiotics that are already resistant. Exogenous aspartate most effectively potentiated tobramycin to lab-evolved and clinical isolated MDR *E. tarda* in *vitro* and in *in vivo* models (fish and mice). Mechanistically, aspartate enhanced tobramycin uptake in MDR *E. tarda* by overcoming efflux pump activity. This effect was mediated through aspartate-induced activation of the pyruvate cycle, which increased the proton motive force via NADH generation and enhanced membrane permeability. Our findings demonstrate that the aspartate-tobramycin combination represents a promising therapeutic strategy against MDR *E. tarda* infections.

## INTRODUCTION

Antibiotic resistance in bacteria poses a global threat to human health and the sustainable development of animal husbandry. This crisis stems from the misuse and overuse of antibiotic agents, rendering many bacteria insensitive to existing drugs (1, 2). The rise of antibiotic-resistant bacteria, coupled with the stagnation in discovering new antibacterials, has created a pressing challenge (3, 4). Consequently, developing novel control strategies is critical (5, 6).

*Edwardsiella tarda*, an intracellular pathogen, not only causes edwardsiellosis in freshwater fish, leading to significant economic losses (7, 8), but also poses a health risk to humans worldwide (9, 10). Multidrug-resistant (MDR) *E. tarda* exhibits resistance to at least three classes of antimicrobial agents, preventing intracellular antibiotics from reaching bactericidal concentrations. This severely limits treatment options, presenting a major challenge in managing infections caused by such resistant strains. However, if these MDR bacteria could be resensitized to existing antibiotics, they could be controlled without waiting for new drug development.

Recent advances in metabolic reprogramming offer a promising strategy to revert antibiotic-resistant bacteria to a drug-sensitive state. This approach relies on reprogramming metabolites, which are identified by comparing the metabolic states of antibiotic-sensitive and antibiotic-resistant bacteria (5). By altering metabolic pathways using the reprogramming metabolites, bacteria that once “resisted” antibiotics can be induced to “accept” (increase uptake) them, increasing intracellular drug concentrations and enhancing bactericidal efficacy (5, 11). While this approach has been proven effective against lab-evolved resistant strains, its application to clinically isolated pathogens remains crucial.

In this study, we employed metabolic reprogramming to reverse antibiotic resistance in *E. tarda*. Using dynamic metabolomics based on the minimum inhibitory concentration (MIC), we identified aspartate as the key metabolite in lab-evolved tobramycin-resistant *E. tarda* (LTB4-R_TOB_). Exogenous aspartate significantly enhanced tobramycin’s efficacy against both LTB4-R_TOB_ and clinically isolated MDR *E. tarda*. Mechanistically, aspartate increased tobramycin uptake by boosting proton motive force (PMF) and membrane permeability.

## RESULTS

### Metabolic profile of LTB4-R_TOB_

**Fig 1:**
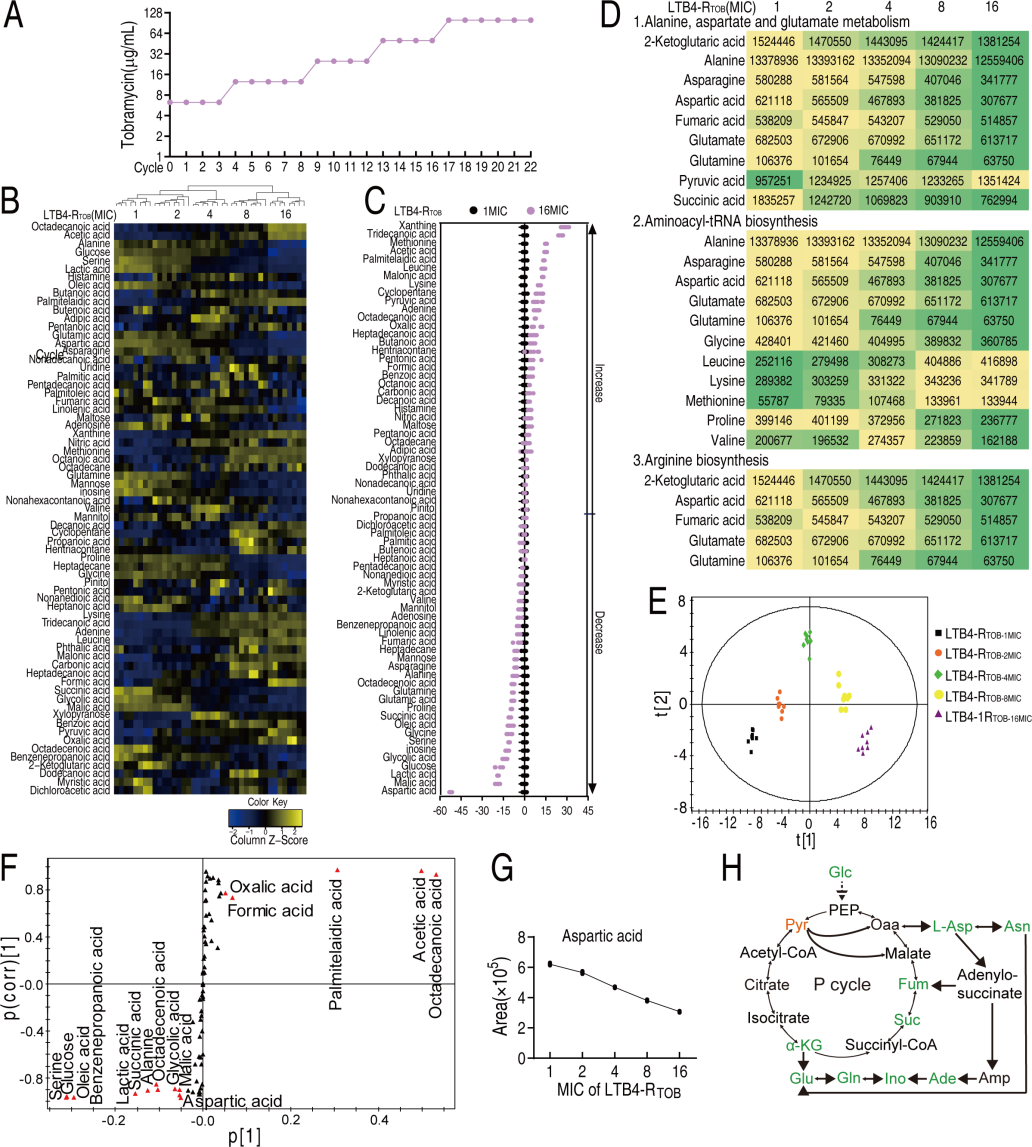
Metabolic profile in LTB4-R_TOB_. (**A**) MIC of LTB4 passaging 1/2 MIC of tobramycin in the indicated cycles. (**B**) Heat map of cluster analysis of differential metabolites in LTB4-R_TOB_. (**C**) *Z*-score plot of LTB4-R_TOB-1MIC_ and LTB4-R_TOB-16MIC_. The data of LTB4-R_TOB-16MIC_ are scaled to the mean and standard deviation of LTB4-R_TOB-1MIC_. (**D**) Analysis of metabolite abundance in pathway enrichment. (**E**) Principal component analysis showing the distribution of LTB4-R_TOB-1MIC_, LTB4-R_TOB-2MIC_, LTB4-R_TOB-4MIC_, LTB4-R_TOB-8MIC_, and LTB4-R_TOB-16MIC_. (**F**) S-plot generated from OPLS-DA for candidate biomarkers. Predictive component *p*[1] and correlation *p* (corr) [1] differentiate these different MIC of LTB4-R_TOB_. (**G**) Aspartate level of these different MICs of LTB4-R_TOB._ (**H**) Diagram showing differential metabolites based on metabolic pathways in LTB4-R_TOB_. α-KG, α-ketoglutarate; Ade, adenine; Amp, adenosine monophosphate; Asn, asparagine; Fum, fumaric acid; Glc, glucose; Gln, glutamine; Glu, glutamic acid; Ino, inosine; L-Asp, aspartic acid; Oaa, oxaloacetic acid; PEP, phosphoenolpyruvate; Suc, succinic acid.

To obtain antibiotic-resistant *E. tarda*, LTB4 was subcultured for 22 cycles in Luria-Bertani (LB) medium with and without 1/2 MIC tobramycin, generating tobramycin-resistant (LTB4-R_TOB_) and tobramycin-sensitive (LTB4-S) strains. MIC was monitored at each cycle. Strains with increasing resistance (1×, 2×, 4×, 8×, and 16× MIC; designated LTB4-R_TOB-1MIC_ to LTB4-R_TOB-16MIC_) from cycles 1, 5, 10, 14, and 18 were selected for gas chromatography–mass spectrometry (GC-MS) metabolomics (Fig. 1A). Forty data sets from four biological replicates (two technical replicates each) showed high reproducibility (correlation coefficients 0.991–0.999, Fig. S1A). The 74 metabolites per sample clustered by MIC dose (Fig. S1B), comprising fatty acids (56.76%), amino acids (22.97%), carbohydrates (9.46%), nucleotides (2.70%), and others (8.11%) (Fig. S1C). Kruskal-Wallis test (*P* < 0.01) identified 69 differentially abundant metabolites across resistance levels, with hierarchical clustering showing LTB4-R_TOB-1MIC_ closest to LTB4-R_TOB-2MIC_, then LTB4-R_TOB-4MIC_, while LTB4-R_TOB-8MIC_ and LTB4-R_TOB-16MIC_ formed a distinct cluster (Fig. 1B). *Z*-score analysis revealed the following comparisons in LTB4-R_TOB-1MIC_: 35 upregulated/34 downregulated metabolites in LTB4-R_TOB-2MIC_; 39 upregulated/30 downregulated in LTB4-R_TOB-4MIC_; 36 upregulated/33 downregulated in LTB4-R_TOB-8MIC_; 35 upregulated/34 downregulated in LTB4-R_TOB-16MIC_, with aspartate being most downregulated (Fig. 1C; Fig. S2). Pathway enrichment showed 22, 20, 17, and 19 enriched pathways in LTB4-R_TOB-2MIC_, LTB4-R_TOB-4MIC_, LTB4-R_TOB-8MIC_, and LTB4-R_TOB-16MIC_, respectively (Fig. S3A), with 13 overlapping pathways where alanine/aspartate/glutamate metabolism showed the strongest MIC-dependent effects (Fig. S3A and B). Most pathway metabolites exhibited gradual MIC-dependent changes (Fig. 1D). Orthogonal partial least squares discriminant analysis (OPLS-DA) separated LTB4-R_TOB-1MIC_/LTB4-R_TOB-2MIC_/LTB4-R_TOB-4MIC_ from _LTB4-RTOB-8MIC_/LTB4-R_TOB-16MIC_ along *t*[1] (Fig. 1E), identifying 16 biomarkers: 10 downregulated (aspartate, octadecanoic acid, alanine, acetic acid, oleic acid, glucose, serine, lactic acid, palmitic acid, and formic acid) and 6 upregulated (phenylpropionic acid, succinic acid, oxalic acid, malic acid, and glycolic acid). Alanine/aspartate/glutamate showed an MIC-dependent reduction (Fig. 1G), with aspartate being the most downregulated and identified as the key reprogramming metabolite, along with its downstream metabolites (Fig. 1G and H).

### Aspartate enhances tobramycin killing in lab-evolved strains

**Fig 2:**
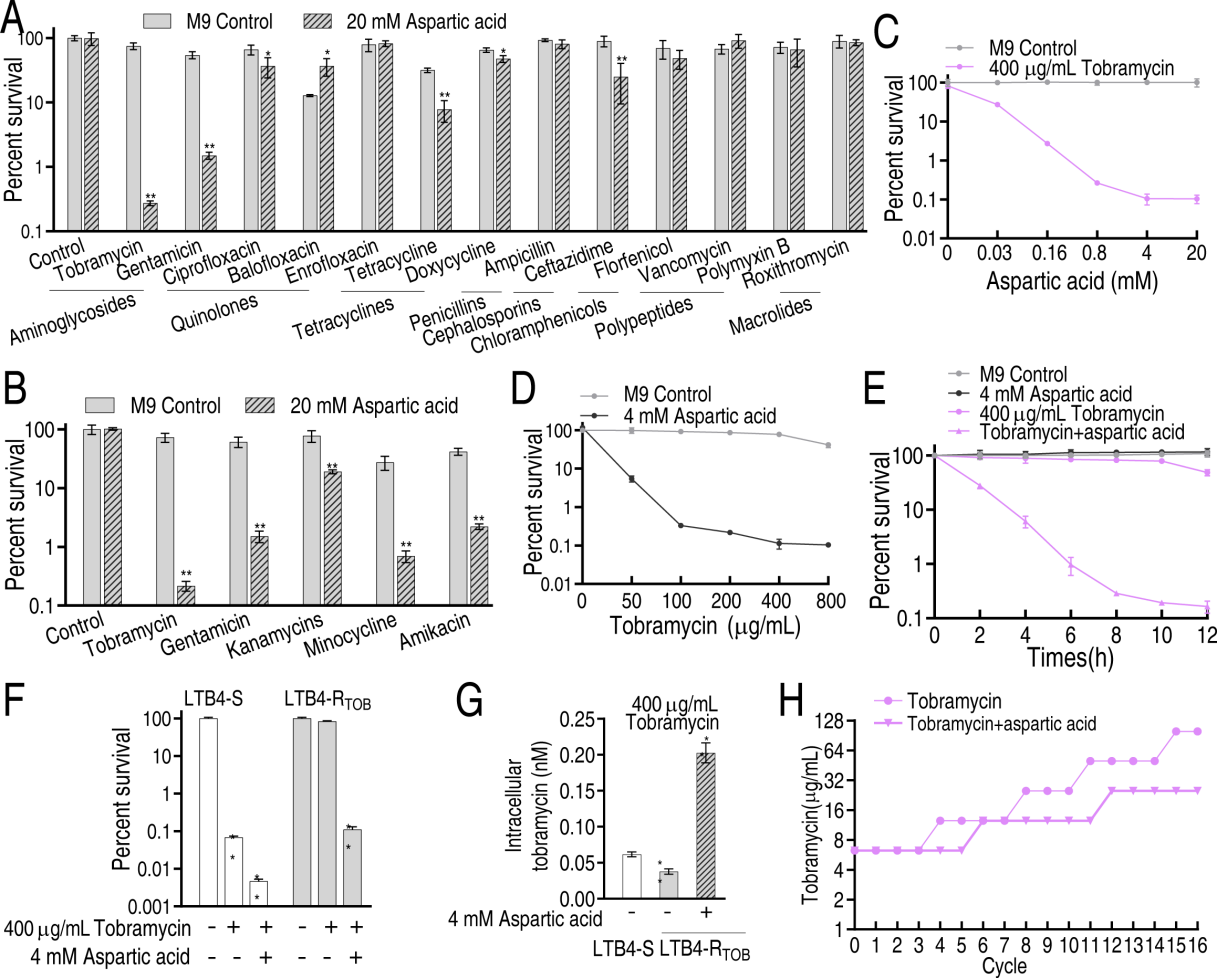
Aspartate potentiates tobramycin to kill LTB4-R_TOB-16MIC_. (**A**) Percent survival of LTB4-R_TOB-16MIC_ in the presence of 20 mM aspartate plus the indicated concentrations of antibiotics as follows: 400 µg/mL tobramycin, 400 µg/mL gentamicin, 15 µg/mL ciprofloxacin, 15 µg/mL balofloxacin, 15 µg/mL enrofloxacin, 100 µg/mL tetracycline, 100 µg/mL doxycycline, 500 µg/mL ampicillin, 400 µg/mL ceftazidime, 50 µg/mL florfenicol, 50 µg/mL vancomycin, 50 µg/mL polymyxin B, and 50 µg/mL roxithromycin. (**B**) Percent survival of LTB4-R_TOB-16MIC_ in the presence of 20 mM aspartate plus the indicated concentrations of aminoglycosides as follows: 400 µg/mL tobramycin, 400 µg/mL gentamicin, 400 µg/mL kanamycin, 400 µg/mL minocycline, and 400 µg/mL amikacin. (**C**) Percent survival of LTB4-R_TOB-16MIC_ in the indicated concentrations of aspartic acid and 400 µg/ mL tobramycin. (**D**) Percent survival of LTB4-R_TOB-16MIC_ in the indicated concentrations of tobramycin and 4 mM aspartic acid. (E) Percent survival of LTB4-R_TOB-16MIC_ in the indicated incubation time plus 400 µg/ mL and 4 mM aspartate. (**F**) Percent survival of LTB4-S and LTB4-R_TOB-16MIC_ in the absence or presence of aspartate or/and tobramycin. (**G**) Intracellular tobramycin of LTB-4 and LTB4-R_TOB_ in the presence of tobramycin or plus aspartate. (**H**) MIC of LTB4 in the absence or presence of tobramycin or tobramycin plus aspartate. Data are mean ± SEM from three biological replicates. *P < 0.05, **P < 0.01.

Key biomarkers can potentiate antibiotic efficacy, and to identify which antibiotics aspartate enhances, we tested 15 antibiotics from 8 classes against LTB4-R_TOB-16MIC_ with or without aspartate. Aspartate significantly improved the killing activity of tobramycin, gentamicin, ciprofloxacin, tetracycline, ceftazidime, and doxycycline compared to monotherapy but showed no synergy with enrofloxacin, ampicillin, florfenicol, vancomycin, polymyxin B, or roxithromycin. Among these, aminoglycosides (tobramycin and gentamicin) showed the strongest enhancement (Fig. 2A). To identify the optimal aspartate-potentiated aminoglycoside, we tested five candidates (tobramycin, gentamicin, kanamycin, minocycline, and amikacin) with aspartate, revealing a bacterial viability gradient: tobramycin < gentamicin < minocycline < amikacin < kanamycin (Fig. 2B), leading to tobramycin’s selection for further study. Aspartate enhanced tobramycin killing dose dependently, peaking at 4 mM aspartate (Fig. 2C), while synergy increased with tobramycin concentrations (50–400 μg/mL) before plateauing (400–800 μg/mL) (Fig. 2D). Time-course experiments showed significantly improved killing efficiency between 2 and 8 h that stabilized from 8 to 12 h (Fig. 2E), establishing optimal conditions as 4 mM aspartate, 400 µg/mL tobramycin, and 8 h incubation. This synergistic effect was effective against both LTB4-S and LTB4-R_TOB-16MIC_ (Fig. 2F), with intracellular tobramycin concentration confirmed as the killing determinant in LTB4-R_TOB-16MIC_ (Fig. 2G). To determine whether aspartate delays the development of tobramycin resistance, LTB4 was serially passaged for 16 cycles in a medium containing 1/2 MIC tobramycin, either with or without aspartate supplementation. The MIC was assessed after each cycle. Results demonstrated a gradual increase in MIC for LTB4 in both treatment conditions over successive passages. However, the aspartate-supplemented group exhibited lower MIC values (25 µg/mL) compared to the non-supplemented group (100 µg/mL) (Fig. 2H). These findings indicate that aspartate delays the emergence of tobramycin resistance in LTB4.

### Aspartate potentiates tobramycin killing in clinically isolated MDR strains

**Fig 3:**
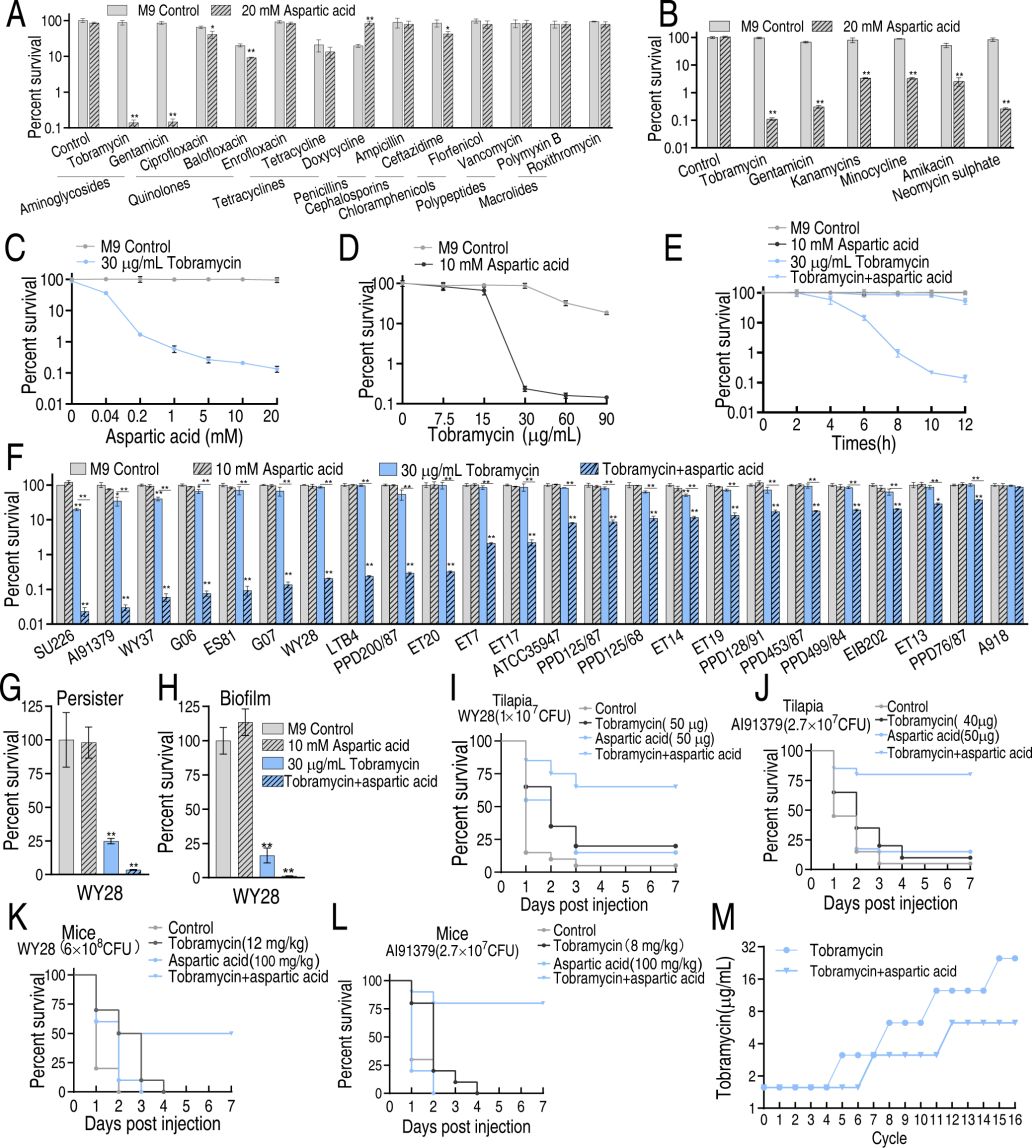
Aspartate potentiates tobramycin to kill WY28. (**A**) Percent survival of WY28 in the presence of 20 mM aspartate plus the indicated concentrations of antibiotics as follows: 30 µg/mL tobramycin, 30 µg/mL gentamicin, 15 µg/mL ciprofloxacin, 15 µg/mL balofloxacin, 15 μg/mL enrofloxacin, 100 µg/mL tetracycline, 100 µg/mL doxycycline, 500 µg/mL ampicillin, 400 µg/mL ceftazidime, 50 µg/mL florfenicol, 50 µg/mL vancomycin, 50 µg/mL polymyxin B, and 50 µg/mL roxithromycin. (**B**) Percent survival of WY28 in the presence of 20 mM aspartate plus the indicated concentrations of aminoglycosides as follows: 30 µg/mL tobramycin, 30 µg/mL gentamicin, 50 µg/mL kanamycin, 50 µg/mL minocycline, 100 µg/mL amikacin, and 50 µg/mL neomycin. (**C**) Percent survival of WY28 in the indicated concentrations of aspartate and 30 µg/mL tobramycin. (**D**) Percent survival of WY28 in the indicated concentrations of tobramycin and 10 mM aspartate. (**E**) Percent survival of WY28 in the indicated incubation time plus 30 µg/mL tobramycin and 10 mM aspartate. (**F**) Percent survival of 24 clinically isolated *E. tarda* in the absence or presence of 30 µg/mL tobramycin or/and 10 mM aspartate. (**G**) Percent survival of WY28 persister in the absence or presence of 30 µg/mL tobramycin or/and 10 mM aspartate. (**H**) Percent survival of WY28 biofilm in the absence or presence of 30 µg/mL tobramycin or/and 10 mM aspartate. (**I**) Percent survival of mice infected with WY28 in the absence or presence of 50 µg tobramycin or/and 50 µg aspartate/fish. (**J**) Percent survival of mice infected with A191379 in the absence or presence of 40 µg tobramycin or/and 50 µg aspartate/fish. (**K**) Percent survival of mice infected with WY28 in the absence or presence of 100 mg/kg tobramycin or/and 12 mg/kg aspartate. (**L**) Percent survival of mice infected with A191379 in the absence or presence of 100 mg/kg tobramycin or/and 8 mg/kg aspartate. (**M**) MIC of WY28 in the absence or presence of tobramycin or tobramycin plus aspartate. Data are mean ± SEM from three biological replicates. *P < 0.05, **P < 0.01.

The aspartate-potentiated killing was further carried out in clinical MDR isolates. We first established optimal killing conditions using MDR strain WY28, testing eight antibiotic classes: aminoglycosides (tobramycin and gentamicin), fluoroquinolones (ciprofloxacin, balofloxacin, and enrofloxacin), tetracyclines (tetracycline and doxycycline), penicillins (ampicillin), cephalosporins (ceftazidime), chloramphenicols (florfenicol), polypeptides (vancomycin and polymyxin B), and macrolides (roxithromycin). Aspartate enhanced bactericidal activity against WY28 by 385.48-fold (tobramycin), 213.75-fold (gentamicin), and 1.11- to 2.18-fold for other antibiotics (Fig. 3A), with maximal potentiation observed for aminoglycosides. Expanded testing of six aminoglycosides revealed enhancement factors of 392.86 (tobramycin), 317.92 (neomycin), 220.93 (gentamicin), and 20.14–27.17 for others (Fig. 3B), confirming tobramycin as the optimal candidate. Dose-response studies established optimal conditions for WY28: bactericidal efficiency increased with aspartate concentration (0.04–20.0 mM), peaking at 10 mM (Fig. 3C). Synergy was concentration dependent for tobramycin (Fig. 3D) and time dependent, reaching maximal effect at 10 h (Fig. 3E). The optimized regimen (10 mM aspartate + 30 µg/mL tobramycin × 10 h) achieved 395.52-fold greater killing than tobramycin alone. This combination showed broad efficacy against 23 out of 24 clinical MDR *E. tarda* strains, with 183.33- to 1,117.65-fold reduction in 10 strains and 2.71- to 40.00-fold in 13 others except for A918 (Fig. 3F). When tobramycin increased, A918 viability also decreased (Fig. S4A). Notably, aspartate enhanced tobramycin’s activity against persistent populations, showing 7.08-fold improved persister killing (Fig. 3G; Fig. S4B and C) and 12.59-fold better biofilm clearance (Fig. 3H). *In vivo* studies demonstrated clinical relevance: in WY28- and AI91379-infected tilapia (1 × 10^7^ CFU), combination therapy maintained 70% survival at day 3 vs 15%–20% for monotherapies (Fig. 3I and J). Mouse models showed similar enhancement: for WY28 infection (6 × 10^8^ CFU), combination therapy yielded 50% survival vs complete mortality in control groups by day 4 (Fig. 3K). Against AI91379 (2.7 × 10^7^ CFU), 80% survival was maintained vs complete mortality in controls (Fig. 3L). To investigate whether aspartate delays the development of tobramycin resistance, WY28 was serially passaged for 16 cycles in the medium containing subinhibitory concentrations (1/2 MIC) of tobramycin, either with or without 10 mM aspartate supplementation. MIC was determined after each passage cycle. While tobramycin resistance progressively increased in both conditions, the aspartate-supplemented cultures exhibited significantly lower MIC values (6.25 µg/mL) compared to the non-supplemented control (25 µg/mL) (Fig. 3M). These results demonstrate that aspartate supplementation attenuates the development of tobramycin resistance in WY28.

### The pharmacokinetics of intracellular tobramycin concentration in WY28 with and without aspartate

**Fig 4:**
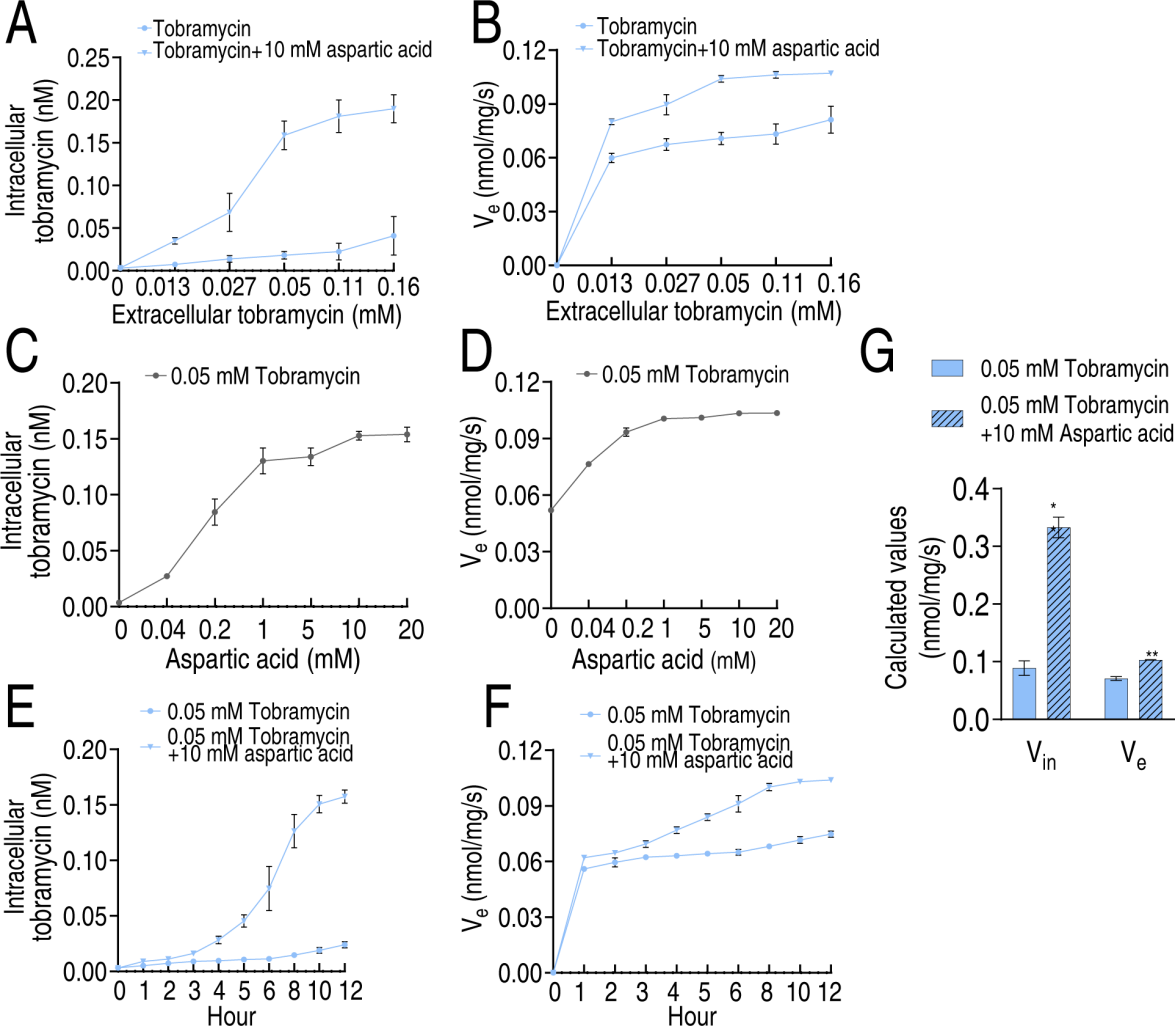
Influx and efflux of WY28 in the absence or presence of aspartate plus tobramycin. Influx (**A**) and efflux (**B**) of WY28 in the indicated concentrations of extracellular tobramycin with or without 10 mM aspartate. Influx (**C**) and efflux (**D**) of WY28 in the indicated concentrations of extracellular aspartate with 0.5 mM tobramycin. Influx (**E**) and efflux (**F**) of WY28 in the indicated incubation time and 0.05 mM tobramycin and 10 mM aspartate. (**G**) Calculated values (nmol/mg/s) of V_in_ and *V*_*e*_ in WY28 at *C*_0_ = MIC. Data are mean ± SEM from three biological replicates. *P < 0.05, **P < 0.01.

The pharmacokinetics of intracellular tobramycin concentration in WY28 with and without aspartate was investigated to determine whether aspartate enhances bacterial uptake of tobramycin. Pharmacokinetic analysis across tobramycin concentrations (0.013–0.16 mM) revealed aspartate significantly increased intracellular tobramycin accumulation (Fig. 4A). While tobramycin efflux increased with concentration alone, aspartate treatment caused efflux to peak at 0.05 mM and plateau up to 0.16 mM (Fig. 4B). Dose-response studies (0.04–20.0 mM aspartate with 0.05 mM tobramycin) showed aspartate concentration dependently enhanced tobramycin uptake (Fig. 4C), with low aspartate concentrations (0.04–1.0 mM) moderately increasing efflux (Fig. 4D). Time-course experiments demonstrated an 8.69-fold increase at 10 h with aspartate (Fig. 4E), accompanied by sustained efflux enhancement (Fig. 4F). At WY28’s MIC (6.25 µg/mL), aspartate increased tobramycin influx (V_in_) from 0.09 to 0.33 nmol/mg/s (3.74-fold) and efflux (*V*_*e*_) from 0.07 to 0.10 nmol/mg/s (1.46-fold) (Fig. 4G), resulting in an 8.72-fold net increase in intracellular tobramycin concentration. These findings demonstrate aspartate primarily enhances tobramycin uptake despite modestly increasing efflux.

### Aspartate-reprogramming metabolomics

**Fig 5:**
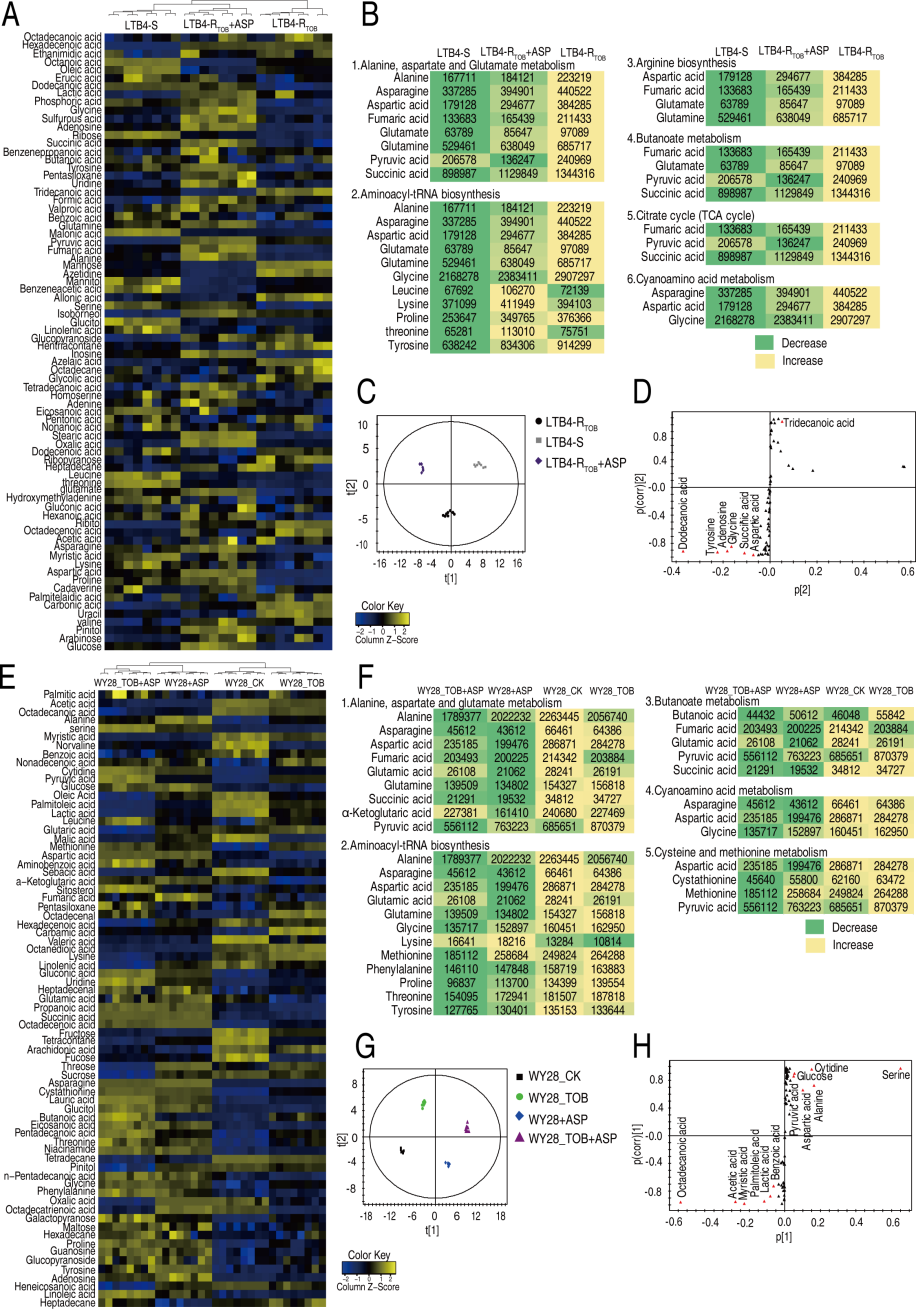
Aspartate reprograms metabolomes of LTB4-R_TOB-16MIC_ and WY28. (**A**) Heatmap of unsupervised hierarchical clustering of metabolites (row) in LTB4-R_TOB-16MIC_. Yellow and blue indicate increase and decrease of the metabolites scaled to mean and standard deviation of row metabolite level, respectively (see color scale). (**B**) Metabolite abundance changes with the color blocks in enriched pathways in LTB4-R_TOB-16MIC_. (**C**) OPLS-DA analysis of MSSA and MRSA using SIMCA + 12.0 LTB4-R_TOB-16MIC_. Each dot represents one technical replicate. (**D**) S-plot generated by OPLS-DA identifies differential metabolites based on *t*[1] in panel A of LTB4-R_TOB-16MIC_. Each triangle represents an individual metabolite, where potential biomarkers are highlighted with red, which is greater than or equal to 0.05 and 0.5 for absolute value of covariance *P* and correlation *P*(corr), respectively. Otherwise, the triangle is marked black. (**E**) Heatmap of unsupervised hierarchical clustering of metabolites (row) in WY28. Yellow and blue indicate increase and decrease of the metabolites scaled to mean and standard deviation of row metabolite level, respectively (see color scale). (**F**) Metabolite abundance changes with the color blocks in enriched pathways WY28. (**G**) OPLS-DA analysis of MSSA and MRSA using SIMCA + 12.0 in WY28. Each dot represents one technical replicate. (**H**) S-plot generated by OPLS-DA identifies differential metabolites based on *t*[1] in panel E of WY28. Each triangle represents an individual metabolite, where potential biomarkers are highlighted with red, which is greater than or equal to 0.05 and 0.5 for absolute value of covariance *P* and correlation *P*(corr), respectively. Otherwise, the triangle is marked black.

Aspartate-reprogramming metabolomics was performed in both lab-evolved LTB4 and clinically MDR WY28. To understand the impact of exogenous aspartate on the metabolic state of LTB4-R_TOB-16MIC_, GC-MS-based programming metabolomics was performed to characterize differential metabolite abundances in LTB4-R_TOB-16MIC_ with aspartate compared to both LTB4-R_TOB-16MIC_ without aspartate and LTB4-S. Technical replicates showed high reproducibility (correlation coefficient ≥0.9988, Fig. S5A). Among 76 identified metabolites per sample (Fig. S5B), 70 showed differential abundance across LTB4-S, LTB4-R_TOB_-_16MIC_, and LTB4-R_TOB-16MIC_ + aspartate. Hierarchical clustering grouped LTB4-S with LTB4-R_TOB-16MIC_ + aspartate, separate from LTB4-R_TOB-16MIC_ (Fig. 5A). Metabolite classes included lipids (40.79%), amino acids (19.74%), carbohydrates (19.74%), nucleotides (6.57%), and others (13.16%) (Fig. S5C). *Z*-score analysis revealed 40 upregulated and 30 downregulated metabolites in LTB4-S, and 47 upregulated/24 downregulated metabolites in LTB4-R_TOB-16MIC_ + aspartate vs LTB4-R_TOB-16MIC_ (Fig. S5D). Pathway enrichment identified 23 and 20 altered metabolic pathways in LTB4-S and LTB4-R_TOB-16MIC_ + aspartate, respectively, vs LTB4-R_TOB-16MIC_ (Fig. S6A), with 15 overlapping pathways. The alanine/aspartate/glutamate pathway was most significantly enriched. Metabolite abundance patterns showed general upregulation in LTB4-S and LTB4-R_TOB_-_16MIC_ + aspartate vs LTB4-R_TOB-16MIC_, with higher levels in the aspartate-treated group (Fig. 5B). OPLS-DA separated groups along *t*[1] (LTB4-S vs others) and *t*[2] (resistant vs sensitive states) (Fig. 5C). S-plot analysis identified seven key biomarkers: aspartic acid, succinic acid, tyrosine, tridecanoic acid, adenosine, dodecanoic acid, and glycine (Fig. 5D).

Parallel metabolomics was performed on WY28 under four conditions: untreated (WY28_CK), aspartate-only (WY28 + ASP), tobramycin-only (WY28_TOB), and combination treatment (WY28_TOB + ASP). High data reproducibility was confirmed (Fig. S7A), with 73 differentially abundant metabolites identified (Fig. 5E). Composition included lipids (43.84%), amino acids (20.55%), carbohydrates (16.43%), nucleotides (8.22%), and others (10.96%) (Fig. S7B). *Z*-scores showed 36 upregulated/37 downregulated in WY28_TOB, 43 upregulated/30 downregulated in WY28 + ASP, and 42 upregulated/31 downregulated in WY28_TOB + ASP vs WY28_CK (Fig. S7C). Pathway analysis revealed 15, 18, and 14 enriched pathways for WY28_TOB, WY28 + ASP, and WY28_TOB + ASP, respectively (Fig. S8A through C), with 11 common pathways (Fig. S8D). Metabolite patterns showed similarities between WY28 + ASP and WY28_TOB + ASP, both distinct from WY28_CK/WY28_TOB (Fig. 5F). OPLS-DA clearly separated groups, with *t*[1] distinguishing WY28-CK/WY28_TOB from WY28 + ASP/WY28_TOB + ASP (Fig. 5G). Key biomarkers included serine, cytidine, glucose, aspartate, alanine, and pyruvate (Fig. 5H). Collectively, the aspartate reprogramming on LTB4-R_TOB-16MIC_ and WY28 suggests that exogenous aspartate primarily promotes alanine, aspartate, and glutamate metabolism (aspartate and alanine); the pyruvate cycle (succinate and pyruvate); and glycine, serine, and threonine metabolism (glycine and serine).

### Identification of exogenous aspartate metabolic flux

**Fig 6:**
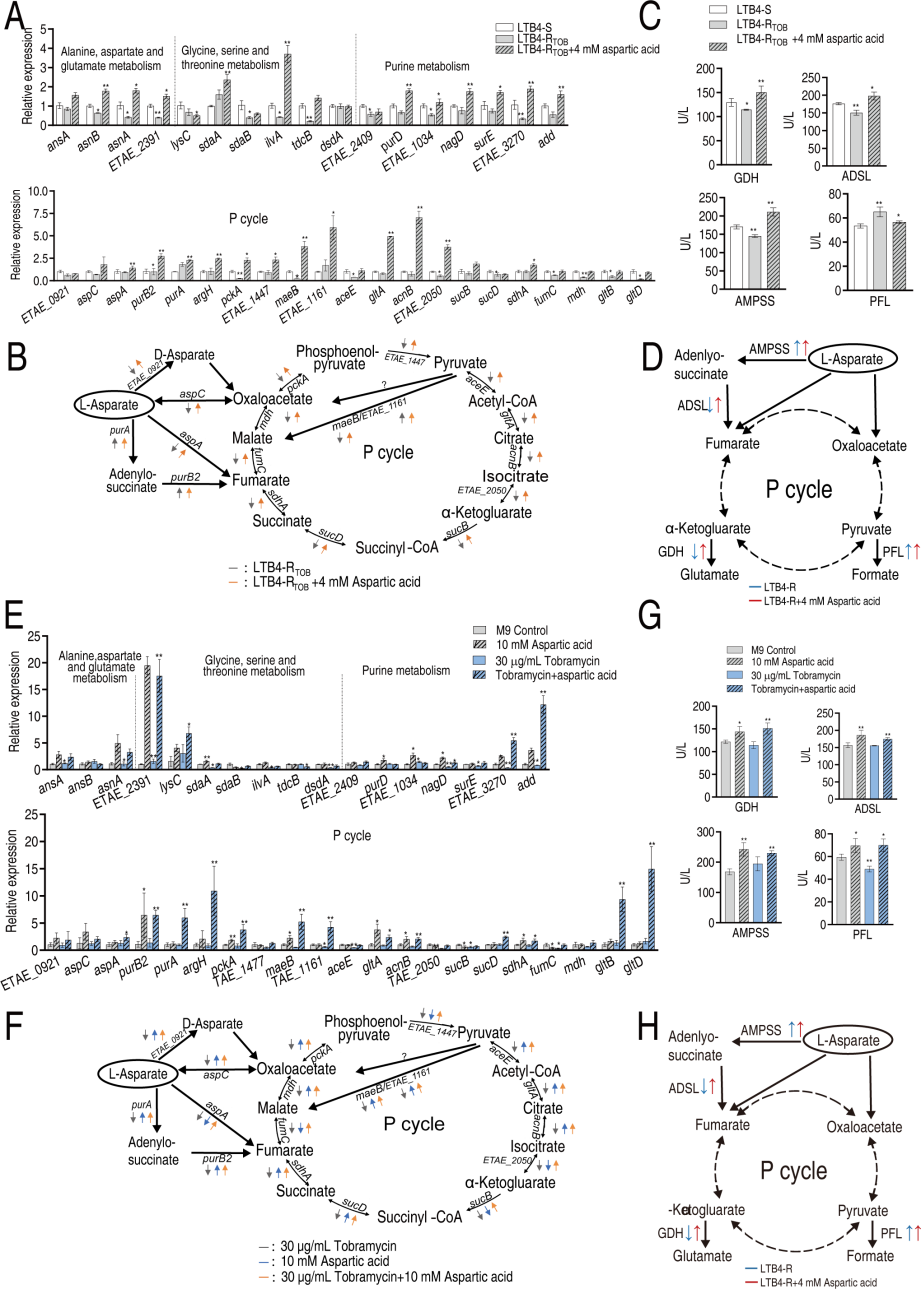
Identification of aspartate metabolic flux. RT-PCR for expression of genes encoding aspartate metabolism-related metabolic pathways in LTB4-R_TOB-16MIC_ with or without 4 mM aspartate and LTB4-S was as a control (**A**) and their summary (**B**). Activity of AMPSS, ADSL, PFL, and GDH in LTB4-R_TOB-16MIC_ with or without 4 mM aspartate and LTB4-S was used as a control (**C**) and their summary (**D**). Quantitative real-time PCR for expression of genes encoding aspartate metabolism-related metabolic pathways in WY28 with or without 10 mM aspartate and LTB4-S was used as a control (**E**) and their summary (**F**). Activity of AMPSS, ADSL, PFL, and GDH in LTB4-R_TOB-16MIC_ with or without 10 mM aspartate and LTB4-S was used as a control (**G**) and their summary (**H**). Data are mean ± SEM from three biological replicates. *P < 0.05, **P < 0.01.

Based on the reprogrammed metabolic states, reverse transcription PCR was used to quantify the expression of genes encoding aspartate metabolism in both lab-evolved LTB4 and clinically MDR WY28. Compared with LTB4-S, LTB4-R_TOB-16MIC_ exhibited downregulated expression levels of 20 genes involved in alanine, aspartate, and glutamate metabolism; glycine, serine, and threonine metabolism; purine metabolism; and the pyruvate cycle, including *asnB* and *asnA* (alanine, aspartate, and glutamate metabolism); ETAE_2391, *sdaB*, and *ilvA* (glycine, serine, and threonine metabolism); *tdcB*, ETAE_2409, *purD*, ETAE_1034, and ETAE_3270 (purine metabolism); and *purB2*, *pckA*, *maeB, aceE*, ETAE_2050, *sucD, fumC*, *mdh*, *gltB*, and *gltD* (pyruvate cycle). Exogenous aspartate restored or even increased the expression of these genes while upregulating most other genes in these pathways (Fig. 6A and B). Key enzyme activities—adenylosuccinate synthase (AMPSS), adenylosuccinate lyase (ADSL), pyruvate formate lyase (PFL), and glutamate dehydrogenase (GDH)—were measured, with AMPSS catalyzing GTP-dependent condensation of inosine monophosphate and L-aspartate to form adenylosuccinate, ADSL mediating cleavage of adenylosuccinate into adenosine monophosphate and fumarate, as well as SAICAR into AICAR and fumarate, PFL reversibly converting pyruvate and CoA into acetyl-CoA and formate, and GDH catalyzing reversible oxidative deamination of L-glutamate to α-ketoglutarate and ammonia. LTB4-R_TOB-16MIC_ showed lower AMPSS, ADSL, and GDH activity but higher PFL activity than LTB4-S, whereas aspartate increased AMPSS, ADSL, and GDH activities in LTB4-R_TOB-16MIC_ beyond LTB4-S levels while normalizing PFL activity (Fig. 6C and D). Replicating this approach in WY28 under different conditions revealed that, compared to the control (no treatment), the tobramycin-only group had reduced expression of 12 genes involved in alanine, aspartate, and glutamate metabolism; glycine, serine, and threonine metabolism; purine metabolism; and the pyruvate cycle, including *sdaA*, *sdaB*, and *ilvA* (glycine, serine, and threonine metabolism) and *nagD*, *surE*, ETAE_3270, *maeB*, ETAE_1161, *aceE*, ETAE_2050, *sucB*, and *fumC* (pyruvate cycle). The aspartate-only group upregulated 9 genes (*asnA*, ETAE_2391, *sdaA*, *purD*, ETAE_1034, *nagD*, ETAE_3270, *add*, *purB2*, *pckA*, *maeB*, *gltA*, *acnB*, and *sdhA*) while downregulating *sucB* and *fumC*, whereas the tobramycin-aspartate group increased expression of 15 genes, including ETAE_2391 (alanine, aspartate, and glutamate metabolism), *lysC* (glycine, serine, and threonine metabolism), ETAE_3270, *add* (purine metabolism), and *aspA*, *purB2*, *purA*, *argH*, *pckA*, *maeB*, ETAE_1161, *gltA*, *acnB*, *sucD*, *sdhA*, *gltB*, and *gltD* (pyruvate cycle), with only *tdcB* downregulated (Fig. 6E and F). Enzyme activity analysis in WY28 showed that, relative to the control, the tobramycin-only group had decreased PFL activity but unchanged AMPSS, GDH, and ADSL activities; the aspartate-only group increased all four enzyme activities (AMPSS, ADSL, PFL, and GDH); and the tobramycin-aspartate group also elevated these enzyme activities (Fig. 6G and H), with results consistent with corresponding gene expression patterns.

### Downstream metabolites of aspartate synergize with tobramycin to enhance bactericidal efficiency

**Fig 7:**
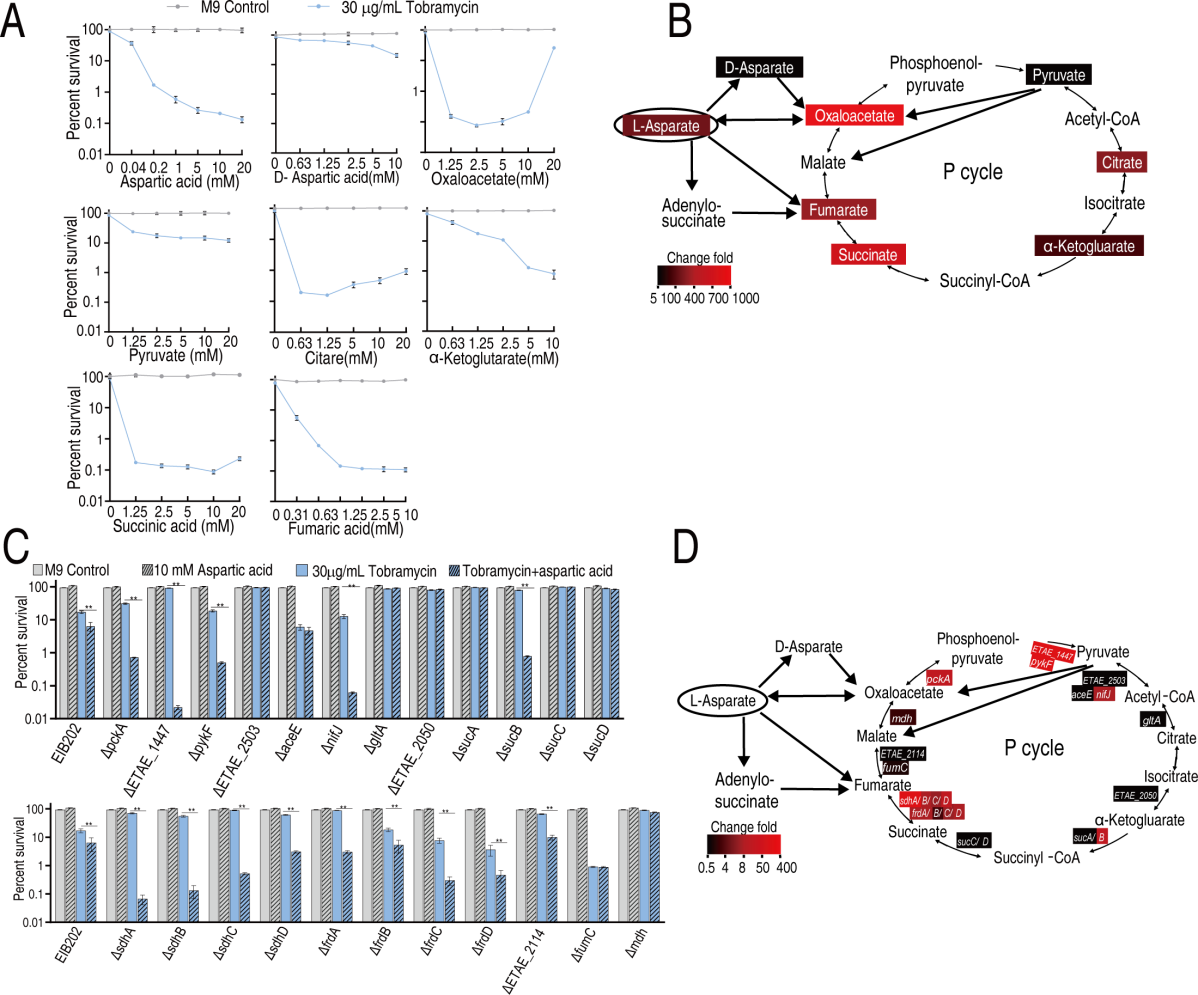
Validation of these key metabolic pathways. (**A**) Percent survival of WY28 in the absence or presence of the indicated concentrations of metabolites plus 30 µg/mL tobramycin. (**B**) Diagram showing the impact of the metabolites on tobramycin killing from data (**A**). (**C**) Percent survival of these indicated mutants in the absence or presence of the indicated concentrations of metabolites plus 30 µg/mL tobramycin. (**D**) Diagram showing the impact of on tobramycin killing from data (**C**). Data are mean ± SEM from three biological replicates. **P < 0.01.

To assess whether aspartate metabolic flux contributes to potentiated killing, we replaced aspartate with its downstream metabolites—D-aspartic acid, pyruvic acid, fumaric acid, succinic acid, aspartic acid, oxaloacetic acid, sodium citrate, and alpha-ketoglutaric acid—and evaluated their ability to enhance tobramycin’s activity against WY28. All metabolites improved tobramycin’s bactericidal efficiency to varying degrees, with oxaloacetic acid, sodium citrate, alpha-ketoglutaric acid, succinic acid, and fumaric acid increasing killing efficacy by over 100-fold (Fig. 7A and B). These findings confirm that alanine, aspartate, and glutamate metabolism; glycine, serine, and threonine metabolism; purine metabolism; and the pyruvate cycle are critical for potentiation, with the pyruvate cycle playing a dominant role as aspartate enters it via glutamate metabolism and glycine/serine/threonine metabolism (12, 13). To further investigate the mechanism, we conducted bactericidal assays using aspartate combined with tobramycin on EIB202 single-gene deletion strains targeting 23 pyruvate cycle-related genes. Compared to EIB202, 11 mutant strains (Δ*aceE*, ΔETAE_2503, Δ*gltA*, ΔETAE_2050, Δ*sucA*, Δ*sucC*, Δ*sucD*, Δ*frdB*, ΔETAE_2114, Δ*fumC*, and Δ*mdh*) exhibited impaired bactericidal efficiency upon aspartate-tobramycin treatment, while deletions of other genes had no significant effect (Fig. 7C and D). These results demonstrate that the pyruvate cycle mediates aspartate’s enhancement of bactericidal activity.

### Aspartate-induced proton motive force mechanism

**Fig 8:**
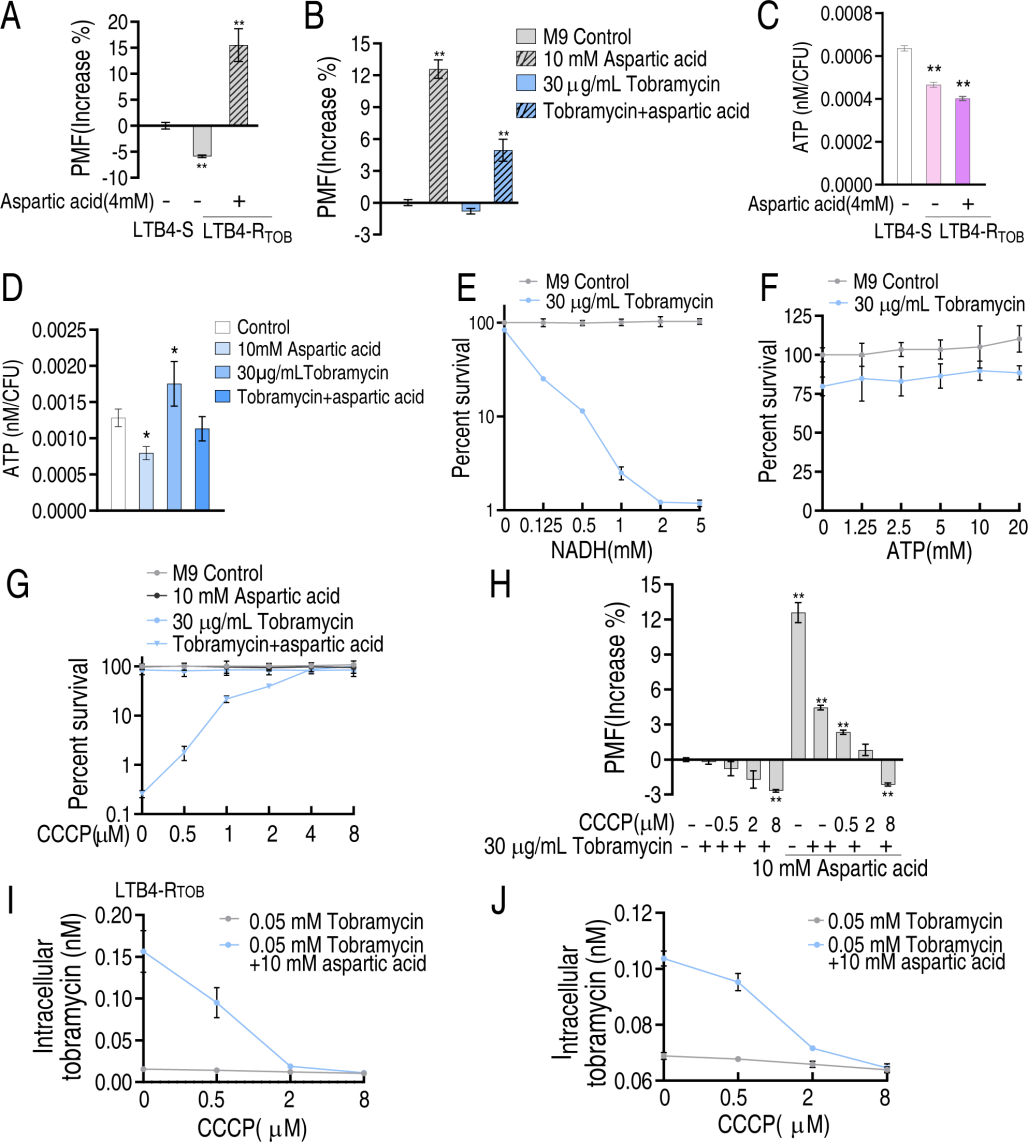
PMF mechanism. PMF of LTB4-R_TOB_ (**A**) and WY28 (**B**) in the absence or presence of aspartate or/and tobramycin. ATP of LTB4-R_TOB_ (**C**) and WY28 (**D**) in the absence or presence of aspartate or/and tobramycin. Percent survival of WY28 in the indicated concentrations of NADH (**E**) or ATP (**F**) plus 30 µg/mL tobramycin. (G) Percent survival of WY28 in the absence or presence of aspartate or/and tobramycin with the indicated concentrations of CCCP. (H) PMF of WY28 in the presence of 30 µg/mL tobramycin with or without 10 mM aspartate plus the indicated concentrations of CCCP. Intracellular tobramycin concentration of LTB4-R_TOB_ (**I**) and WY28 (**J**). Data are mean ± SEM from three biological replicates. *P < 0.05, **P < 0.01.

It has been reported that an enhanced pyruvate cycle increases NADH and ATP production (12). NADH contributes to the PMF, thereby promoting ATP biosynthesis. In our study, PMF was lower in LTB4-R_TOB-16MIC_ than in LTB4-S, but aspartate significantly increased PMF in LTB4-R_TOB-16MIC_ (Fig. 8A). In WY28, PMF levels followed this order: aspartate alone > aspartate plus tobramycin > control ≈ tobramycin alone (Fig. 8B). Interestingly, exogenous aspartate reduced ATP levels in LTB4-R_TOB-16MIC_, which were lower than those in LTB4-S (Fig. 8C). In WY28, ATP levels varied under different conditions: compared to the untreated control (no aspartate or tobramycin), aspartate alone decreased ATP; tobramycin alone increased ATP; and the combination of aspartate and tobramycin had no significant effect (Fig. 8D). To determine whether downstream metabolites mediate this potentiation effect, we replaced aspartate with NADH or ATP to assess their ability to enhance tobramycin killing. NADH potentiated tobramycin activity against WY28 in a dose-dependent manner (Fig. 8E), whereas ATP did not (Fig. 8F). Additionally, WY28 viability increased dose dependently with the protonophore CCCP (Fig. 8G), due to PMF disruption (Fig. 8H). This led to reduced drug uptake in both LTB4-R_TOB-16MIC_ and WY28 (Fig. 8I and J).

### Aspartate enhances bacterial membrane permeability to potentiate tobramycin uptake

**Fig 9:**
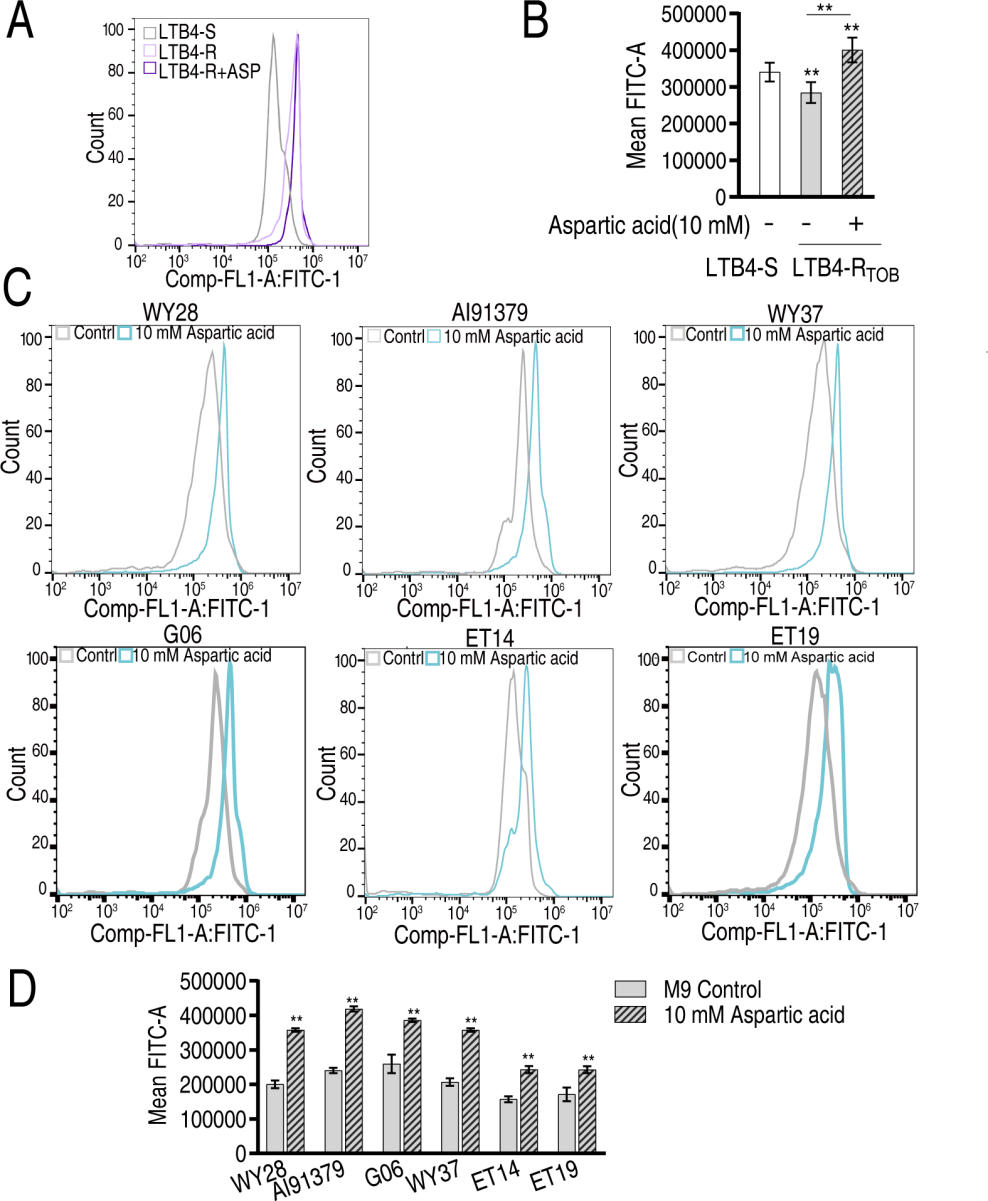
Aspartate reverses the reduced membrane permeability of antibiotic-resistant *E. tarda.* Membrane permeability of LTB4-R_TOB_ in the absence or presence of tobramycin with or without aspartate from raw data (**A**) and summary (**B**). Membrane permeability of clinically isolated MDR-*E. tarda* in the absence or presence of tobramycin with or without aspartate from raw data (**C**) and summary (**D**). Data are mean ± SEM from three biological replicates. **P < 0.01.

Exogenous aspartate significantly enhanced tobramycin uptake (Fig. 4), suggesting improved membrane permeability. To validate this mechanism, we quantitatively assessed membrane permeability in three experimental groups: drug-sensitive LTB4-S, tobramycin-resistant LTB4-R_TOB-16MIC_, and aspartate-treated LTB4-R_TOB-16MIC_. Comparative analysis revealed a distinct permeability gradient: aspartate-treated resistant strains exhibited the highest permeability, exceeding both untreated resistant strains and drug-sensitive controls (LTB4-R_TOB-16MIC_ + aspartate > LTB4-S > LTB4-R_TOB-16MIC_) (Fig. 9A and B). This phenomenon was further confirmed in a panel of six clinically isolated strains, where aspartate supplementation consistently increased membrane permeability across all tested isolates (Fig. 9C and D). These collective results provide conclusive evidence that exogenous aspartate serves as a potent modulator of bacterial membrane permeability.

## DISCUSSION

Metabolic reprogramming is being used to increase antibiotic killing and restore host anti-infective ability (14–19). In the potentiated antibiotic killing, almost all studies focus on lab-evolved strains (20–24), while those targeting clinical isolates are rare. In the present study, reprogramming metabolite aspartate is identified from lab-evolved LTB4-R_TOB_ but applied in both lab-evolved tobramycin-resistant and clinically isolated strains. The antibiotic most potentiated by aspartate is tobramycin. Aspartate effectively promotes tobramycin to kill both lab-evolved and clinically isolated MDR strains. This is because aspartate boosts the P cycle to increase PMF and elevates membrane permeability to promote tobramycin uptake in these strains. The promoted uptake overcomes the dampening by efflux, and intracellular tobramycin concentration is thereby elevated to implement the high killing efficiency. Therefore, this study identifies an ideal reprogramming metabolite that is effective to both lab-evolved and clinically isolated strains and reveals the metabolic reprogramming mechanism that elevates tobramycin uptake. These findings provide a reprogramming metabolite candidate for developing next-generation aminoglycosides (11), which is implemented by an aminoglycoside plus a reprogramming metabolite that promotes drug uptake. Notably, although aspartate enhances the bactericidal effect of tobramycin on both lab-evolved strain and clinical isolates, the optimal conditions for killing, such as tobramycin and aspartate concentrations, differ between them, suggesting that the optimal conditions derived from lab-evolved strains need to be optimized for application to clinical isolates.

Antibiotic-sensitive and antibiotic-resistant bacteria have antibiotic-sensitive and antibiotic-resistant metabolic states, respectively. Key biomarkers representing the metabolic states contribute to the antibiotic resistance or susceptibility, where downregulated biomarkers are selected as reprogramming metabolites (20, 25–27). Since the evolution from an antibiotic-sensitive strain to an antibiotic-resistant strain is a gradual process, biomarkers that reduced with the evolution should be an ideal reprogramming metabolite. To test this, this study carries out a dynamic metabolomics to understand the dynamic changes in metabolic states of LTB4-R_TOB_. Aspartate is identified as the most key reprogramming metabolite because it is the most downregulated metabolite, one of these reduced biomarkers, a metabolite belonging to the most enriched metabolic pathway and reducing with the increasing MIC. Therefore, dynamic metabolomics of evolved strains provides a solid proof to identify the most ideal reprogramming metabolite.

Among the four well-recognized mechanisms of resistance, reduced membrane permeability lowers intracellular antibiotic concentrations by limiting antibiotic uptake. The other three mechanisms target these intracellular antibiotic concentrations, including the expulsion of intracellular antibiotics to the extracellular environment via efflux pumps and the hydrolysis of intracellular antibiotics by β-lactamases, thereby reducing the intracellular antibiotic concentration below the bactericidal threshold (11, 28). However, when uptake exceeds the combined effects of the efflux and hydrolysis, the intracellular antibiotic concentration can exceed the bactericidal threshold to kill the bacteria. Therefore, enhancing antibiotic uptake is considered a promising strategy for the development of next-generation antibiotics (11). The present study shows that aspartate is a reprogramming metabolite that increases tobramycin uptake. This increased uptake is higher than the dampening by efflux pumps (β-lactamases were not analyzed since they do not hydrolyze aminoglycosides), resulting in intracellular antibiotic concentrations above the bactericidal threshold. Therefore, aspartate effectively potentiates tobramycin to kill MDR *E. tarda in vitro* and *in vivo*. These findings provide an effective reprogramming metabolite candidate for next-generation antibiotics.

The mechanism by which aspartate enhances the bactericidal effect of tobramycin is revealed through the combination of reprogramming metabolomics with biochemistry and molecular biology techniques. Through tracking the metabolic flow of aspartate, it is demonstrated that two mechanisms, increasing PMF and enhancing membrane permeability, are involved. This is reasonable, as only the simultaneous action of these two mechanisms can achieve the best effect. The PMF provides the driving force for antibiotic influx into the cell, while membrane permeability provides the pathway for antibiotic influx. Aspartate possesses both of these effects, further demonstrating its potential as a candidate for reprogrammed metabolites in next-generation antibiotics.

Notably, A918 is the only *E. tarda* strain among the tested isolates that exhibits resistance to the combined tobramycin-aspartate treatment. This resistance phenotype likely stems from both its intrinsic high-level tobramycin resistance and its unique metabolomic and membrane properties. While elevated tobramycin resistance correlates with the observed phenotype, the specific contributions of its distinct metabolic profile and membrane characteristics remain to be elucidated. To systematically investigate these resistance mechanisms, we propose employing well-curated strain collections with minimized clinical variability. Such an approach would facilitate comprehensive metabolic profiling and membrane permeability analyses, enabling clearer mechanistic insights.

In summary, a dynamic metabolomics was performed in tobramycin-resistant *E. tarda* LTB4 (LTB4-R_TOB-1MIC_, LTB4-R_TOB-2MIC_, LTB4-R_TOB-4MIC_, LTB4-R_TOB-8MIC_, and LTB4-R_TOB-16MIC_). Aspartate was identified as the ideal reprogramming metabolite. Exogenous aspartate effectively potentiates tobramycin against not only the LTB4-R but also clinically isolated strains. This is because aspartate increases intracellular drug concentration. Aspartate-reprogrammed LTB4-R_TOB-16MIC_ and WY28, a clinically isolated strain, identify exogenous aspartate metabolic flux to enter alanine, aspartate, and glutamate metabolism; the pyruvate cycle; glycine, serine, and threonine metabolism. This leads to the elevation of PMF and the increase of membrane permeability to promote drug uptake. These results indicate that aspartate is an ideal reprogramming metabolite to promote tobramycin killing efficiency.

## MATERIALS AND METHODS

### Bacterial strains

All bacterial strains were obtained from our lab collection. Specifically, strains WY28 and LTB4 were isolated from aquaculture environments, with WY28 kindly provided by Professor Yuanxin Zhang from East China University of Science and Technology and LTB4 by Professor Xiaohua Zhang from Ocean University of China.

### MIC determination in LB medium using broth microdilution method

The MICs were determined following Clinical and Laboratory Standards Institute guidelines (29) with modifications. Overnight cultures were prepared by inoculating three to four isolated colonies from tryptic soy broth (TSB) agar plates into 5 mL LB broth, followed by incubation at 30°C with 200 rpm shaking for 24 h. These saturated cultures were then diluted 1:100 in fresh LB medium and grown to mid-log phase (optical density at 600 [OD_600_] = 0.5) under identical conditions. For microdilution assays, bacterial suspensions were standardized to 5 × 10⁶ CFU/mL in LB broth. Antibiotic solutions (200 µL) were dispensed into the first column of sterile 96-well plates, with 100 µL LB medium added to all remaining wells. Twofold serial dilutions were performed across the plate using a multichannel pipette, leaving the final column as a sterility control (LB medium only). Each test well received 10 µL of bacterial suspension, achieving a final inoculum of 5 × 10⁴ CFU/well in 100 µL total volume. Plates were incubated statically at 30°C for 16 h. The MIC endpoint was determined as the lowest antibiotic concentration showing complete inhibition of visible growth. All determinations were performed in triplicate biological replicates.

### The acquisition of LTB4-R_TOB_

Three to four isolated colonies of LTB4 from TSB agar plates were inoculated into 5 mL LB broth and cultured at 30°C with 200 rpm shaking for 24 h. The culture was then subcultured (1:50, vol/vol) into fresh LB medium containing 1/8× MIC of target antibiotic, with subsequent passages performed every 12 h under identical conditions while progressively increasing antibiotic concentrations (1/4×, 1/2×, and 1× MIC) through successive generations. Prior to each MIC determination, bacterial cells were collected by centrifugation (8,000 rpm, 5 min) and thoroughly washed three times with sterile 0.9% NaCl solution. Resistance development was tracked through periodic MIC testing, with resistance levels expressed as fold changes relative to the parental strain’s baseline MIC.

### GC-MS metabolomic profiling

#### Sample preparation

Three to four single bacterial colonies from a TSB agar plate were inoculated into 50 mL of TSB medium and incubated at 30°C for 24 h. Cells were harvested by centrifugation at 8,000 rpm for 3 min, washed three times with saline (0.9% NaCl), and resuspended in M9 minimal medium to an OD_600_ of 0.2. Metabolites and/or antibiotics were added, and the culture was incubated at 30°C with shaking at 200 rpm for 10 h (optimal conditions determined via preliminary survival rate assays).

#### Metabolite extraction and derivatization

Metabolic activity was quenched by rapid addition of 1:1 80°C pre-chilled 100% methanol (20). Cells were pelleted by centrifugation (8,000 rpm, 3 min, 4°C), washed three times with saline, and resuspended in saline to an OD_600_ of 1.0. For each sample (10 mL suspension), aliquots were transferred to 1.5 mL QSP tubes. Cold methanol (1 mL) containing 10 µL of 0.1 mg/mL ribitol (internal standard) was added to each aliquot. Cells were lysed by sonication (650 W, 35% amplitude, 2 s on/3 s off cycles, 10 min total), followed by centrifugation (12,000 × *g*, 10 min, 4°C) to pellet debris. The supernatant was transferred to fresh tubes and dried in a Labconco vacuum concentrator at 37°C for 3 h. For derivatization, dried metabolites were suspended in 80 µL of 20 mg/mL methoxyamine hydrochloride in pyridine and incubated at 37°C with shaking at 200 rpm for 3 h. Subsequently, 80 µL of N-methyl-N-trimethylsilyltrifluoroacetamide containing 1% trimethylchlorosilane was added, and the mixture was incubated at 37°C for 45 min. Derivatized samples (120 µL) were transferred to gas chromatography vials for analysis.

#### GC-MS analysis

Analysis was performed using an Agilent 7890A gas chromatograph coupled to a 5975C mass spectrometer (Agilent Technologies, USA) (30). Separation was achieved on a DB-5MS capillary column (30 m × 0.25 mm × 0.25 µm) with helium as the carrier gas (1.0 mL/min). The injection volume was 1 µL in splitless mode. The oven temperature program was 70°C (5 min hold), ramped at 2 °C/min to 270°C (5 min hold). Temperatures were 270°C (inlet), 270°C (interface), 230°C (ion source), and 150°C (quadrupole). Mass spectra were acquired in full-scan mode (60–600 *m*/*z*) at 70 eV ionization energy.

#### Analysis of GC-MS data

Raw data were converted to netCDF format using Agilent Chrom Station software (30). Peaks were aligned, deconvoluted, and annotated using the NIST 2008 mass spectral library and Golm Metabolome Database. Internal standard (ribitol)-normalized peak areas were log-transformed and Pareto-scaled prior to analysis. Statistical workflows included differential metabolites: two-tailed Student’s *t*-tests and Wilcoxon rank-sum tests with false discovery rate correction (*P* < 0.05).

#### Multivariate analysis

OPLS-DA and principal component analysis were performed using SIMCA-P+ 12.0 (key metabolites: |*P*(corr)| ≥0.5). Hierarchical clustering and heatmaps were generated using R 3.6.1 (*P* < 0.01). Pathway enrichment was performed using MetaboAnalyst 4.0 (*P* < 0.05 significance threshold). Score normalization was calculated based on control group means and standard deviations.

### Antibiotic bactericidal assays

This assay was performed as described previously (20). Bacterial cultures were harvested by centrifugation at 8,000 rpm for 5 min at 4°C, washed three times with sterile saline (0.9% NaCl), and resuspended in M9 minimal medium supplemented with 10 mM sodium acetate, 1 mM MgSO_4_, and 100 µM CaCl_2_ to an OD_600_ of 0.2. Aliquots were treated with metabolites or/and antibiotics, and the control group without adding substances and antibiotics was set aside, followed by incubation at 30°C with shaking at 200 rpm for 10 h. Post-treatment, cultures were serially diluted in saline (10-fold gradients). For quantification, 5 µL of each dilution was spotted onto TSB agar plates and incubated at 30°C for 18–24 h. Colonies within the 20–200 CFU/spot range were counted. Data were derived from three independent biological replicates.

### Persister assays

This assay was performed as described previously (20). Bacteria were cultured overnight in LB medium at 30°C with shaking at 200 rpm to stationary phase (~6 × 10^9^ CFU/mL). Cells were treated with 5 µg/mL ofloxacin for 2 h at 30°C to eliminate metabolically active cells and enrich persister populations. Persisters were validated by extending ofloxacin treatment to 8 h and escalating the concentration to 40 µg/mL, with no further reduction in viability observed. Enriched persisters were washed twice with sterile saline (0.9% NaCl) and resuspended in M9 minimal medium supplemented with aspartate to an OD_600_ of 0.2. Metabolites and/or antibiotics were added as specified, followed by incubation at 30°C with shaking at 200 rpm for up to 10 h. At designated time points, 100 µL aliquots were collected, washed to remove residual drugs, and serially diluted in saline. Survival rates were quantified by spot plating 5 µL of dilutions onto TSB agar plates. Colonies were enumerated after 24 h incubation at 30°C, with survival calculated.

### Biofilm assays

This assay was performed as described previously (20). Bacterial biofilms were established on PE50 catheter surfaces by incubating strains in TSB medium at 30°C for 72 h under static conditions. The medium was refreshed every 24 h to maintain nutrient availability. Catheters were gently rinsed with sterile saline (0.9% NaCl) to remove planktonic cells and transferred to M9 minimal medium supplemented with aspartate. Biofilms were treated with metabolites and/or antibiotics at 30°C with shaking at 200 rpm for 10 h. These treated samples were serially diluted (10-fold gradients) in saline, and 5 µL of each dilution was spot-plated onto TSB agar. Plates were incubated at 30°C for 24 h. Colonies within the 20–200 CFU/spot range were counted, and viability was calculated. Biofilm integrity was confirmed via crystal violet staining prior to treatment. Untreated biofilm controls and planktonic cell controls were included to distinguish biofilm-specific tolerance.

### Systematic infection and therapy in a tilapia model

This assay was performed as described previously (31). Three to four single bacterial colonies were cultured in 5 mL of TSB at 30°C with shaking at 200 rpm for 24 h until reaching saturation. The bacterial suspension was adjusted to an OD_600_ of 1.0 using fresh TSB medium. Cells were harvested by centrifugation at 5,000 rpm for 3 min, and the supernatant was discarded. The pellet was resuspended in fresh TSB and washed twice under identical centrifugation conditions. A 5 µL aliquot of the prepared bacterial suspension was intraperitoneally injected into Nile tilapia (3–5 cm in length) using a parallel insertion technique from the caudal to cranial direction. The experiment comprised four groups, with 20 fish per group. At 1 h post-infection, therapeutic agents and/or antibiotics were administered via intramuscular injection. Mortality rates were monitored and recorded at 24, 48, 96, 120, 144, 168, and 192 h post-treatment.

### Systematic infection and therapy in a murine model

This assay was performed as described previously (30). A single bacterial colony was cultured in 5 mL of TSB at 30°C with shaking at 200 rpm for 24 h until reaching saturation. The bacterial suspension was adjusted to an OD_600_ of 1.0 using fresh TSB medium. Cells were harvested by centrifugation at 5,000 rpm for 3 min, and the supernatant was discarded. The pellet was resuspended in fresh TSB and washed twice under identical centrifugation conditions. A 300 µL aliquot of the prepared bacterial suspension (see Results for injection volume details) was intraperitoneally injected into BALB/c mice (6–8 weeks old, approximately 20 g) using a parallel insertion technique. The injection site was located 0.5 cm medial to the femoral root, with the needle inserted from a caudal to cranial direction. The experiment comprised 4 groups, with 10 mice per group. At 1 h post-infection, therapeutic agents and/or antibiotics were administered via intramuscular injection. For intramuscular delivery, the needle was inserted parallel to the thigh muscle (half the needle length depth) from a caudal to cranial direction. The plunger was slowly depressed to deliver the solution, followed by rotation of the needle at 180°, holding for 1 s, and withdrawing gently. Mortality rates were monitored and recorded at 24, 48, 96, 120, 144, 168, and 192 h post-treatment.

### Quantitative real-time PCR

Quantitative real-time PCR (qRT-PCR) was carried out as described previously (32). Total RNA was extracted from harvested bacterial cells using TRIzol reagent (Invitrogen, USA). RNA purity and concentration were quantified using a NanoDrop 2000 spectrophotometer (Thermo Fisher Scientific, USA). Reverse transcription was performed with the Evo M-MLV RT Kit (Accurate Biotechnology, China) using 1 µL of total RNA, following the manufacturer’s protocol. qRT-PCR assay was conducted in 384-well plates on a Light Cycler 480 II system (Roche, Germany). Each reaction (10 µL total volume) contained 5 µL of 2 × SYBR Green Pro Taq HS Premix (Accurate Biotechnology), 0.2 µL each of forward and reverse primers (10 µM; see Table S1), 2.6 µL nuclease-free water, and 2 µL cDNA template. Cycling conditions included an initial denaturation at 95°C for 30 s, followed by 40 cycles at 95°C for 5 s, 58°C for 30 s (annealing/extension), and fluorescence acquisition at 70°C for 1 s per cycle. A melting curve analysis (95°C–65°C, 0.5 °C/s ramp rate) was performed to confirm primer specificity. Data were analyzed using LightCycler 480 Software (v.1.5.1) with the ΔΔCt method. Gene expression levels were normalized to the housekeeping gene, *rpoB*, and expressed as fold changes relative to the untreated control group. Four biological replicates were included for both test and control conditions.

### Measurement of enzyme activity

Based on the bacterial survival rate assay protocol, cultures were treated with specific metabolites and/or antibiotics, alongside an untreated control group (no additives or antibiotics), and incubated at 30°C with shaking at 200 rpm for 10 h (optimal conditions determined from survival rate experiments). Post-incubation, cells were harvested by centrifugation at 8,000 rpm for 3 min, washed twice with sterile saline (0.9% NaCl), and resuspended in saline to an OD_600_ of 1.0. Aliquots (10 mL) of the resuspended culture were transferred to 1.5 mL microcentrifuge tubes, and cells were further resuspended in 400 µL saline and sonicated at 650 W (35% amplitude) using a pulsing cycle of 2 s on and 3 s off for 3 min. The lysate was centrifuged at 12,000 rpm for 10 min at 4°C, and the supernatant was collected. Protein concentration was quantified using a BCA Protein Assay Kit (Beyotime Biotechnology, China) and adjusted to 3 mg/mL. All reagents were equilibrated to room temperature (20°C–25°C), and the 20× wash buffer was diluted with distilled water at a 1:20 ratio prior to use. All assays were performed in triplicate (three biological replicates). For intracellular enzyme activity assays, 10 µL of the 3 mg/mL supernatant was analyzed for ADSL using the Microbial ADSL ELISA Kit (MEIMIAN, China), AMPSS with the AMPSS ELISA Kit (MEIMIAN), GDH with the GDH ELISA Kit (MEIMIAN), and PFL with the PFL ELISA Kit (MEIMIAN, China), following the respective manufacturer’s protocols, with all steps performed in triplicate to ensure reproducibility.

### Intracellular tobramycin efflux kinetic

Bacterial cultures were resuspended in M9 minimal medium and adjusted to an OD_600_ of 0.2. Specific metabolites and/or antibiotics were added to designated groups, with an untreated control group (no additives or antibiotics) included. All groups were incubated at 30°C with shaking at 200 rpm for 10 h. After incubation, cells were pelleted by centrifugation at 8,000 rpm for 3 min, washed twice with sterile saline, and resuspended in saline to an OD_600_ of 1.0. Aliquots (1 mL) were transferred to 1.5 mL microcentrifuge tubes. Cells were further resuspended in 400 µL saline and sonicated at 650 W (35% amplitude) with a cycle of 2 s on and 3 s off for 3 min. The lysate was centrifuged at 12,000 rpm for 10 min at 4°C, and the supernatant was collected. Protein concentration was quantified using a BCA Protein Assay Kit (Beyotime Biotechnology, P009) and adjusted to 3 mg/mL. Intracellular tobramycin levels were measured using 50 µL of the 3 mg/mL supernatant according to the Jianglai Biotechnology Tobramycin ELISA Kit (JL53907). The efflux rate (*V*_*e*_) was calculated using the Michaelis-Menten equation: ‌*V*_*e*_ = Vemax × (*C*_*p*_)^*h*^ / [(*K*_0.5_)^*h*^ + (*C*_*p*_)^*h*^]‌, where Vemax (maximal efflux rate) = 0.37 nmol/mg/s; *K*_0.5_ (half-maximal velocity drug concentration) = 0.94 µM; *h* (Hill coefficient) = 0.23; and *C*_*p*_ (intracellular tobramycin concentration) was determined via the enzyme-linked immunosorbent assay method. In the absence of aspartate, the influx rate (V_in_) was calculated as V_in_ = *P* × *A* × (*C*_0_ – *C*_*p*_)‌,where *P* (permeability coefficient) = 0.28 × 10⁻⁵ cm/s; *A* (bacterial surface area) = 10^3^ cm²/mg; and *C*_0_ (MIC of tobramycin against LTB4-R_TOB_) = 100 µg/mL (equivalent to 0.18 mM). In the presence of aspartate, V_in_ was modified to: V_in_ = *P* × *A* × (*C*_*p*_ without aspartate / *C*_*p*_ with aspartate) × (*C*_0_ – *C*_*p*_). These parameters were from a previous report with a modification (33).

### PMF measurement

In the determination of bacterial cell membrane PMF, the experimental procedure was prepared according to bacterial survival rate assays. Bacterial cultures were harvested by centrifugation at 8,000 rpm for 5 min at 4°C, washed three times with sterile saline (0.9% NaCl), and resuspended in M9 minimal medium supplemented with 10 mM sodium acetate, 1 mM MgSO4, and 100 µM CaCl_2_ to an OD_600_ of 0.2. Specific metabolites, antibiotics, and CCCPs were added to respective treatment groups, with an untreated control group (no additives or antibiotics) established. Cultures were incubated at 30°C with shaking at 200 rpm for 10 h. After incubation, cultures were washed three times with sterile saline (0.9% NaCl) and diluted 10- to 100-fold, and 1 mL aliquots were dispensed into microcentrifuge tubes. DiOC_2_ (3 mM, 10 µL) was added under light-protected conditions, followed by incubation at 30°C with shaking at 200 rpm for 0.5 h in the dark. Cells were washed twice with M9 medium after incubation, and flow cytometry was performed to measure fluorescein isothiocyanate (FITC) fluorescence intensity (P2 channel) and PC5.5 fluorescence intensity (P3 channel). During detection, the cell flow rate was maintained at 400–600 CFU/min, with a total recorded count of 10,000 CFU. PMF was calculated using the formula log(10 × PC5.5 / FITC) + 1.5. The PMF of experimental groups was normalized to 100% for the control group, and relative PMF differences were determined.

### Membrane permeability measurement

Membrane permeability measurement was performed by harvesting bacterial cultures through centrifugation at 8,000 rpm for 5 min at 4°C, followed by three washes with sterile saline (0.9% NaCl) and resuspension in M9 minimal medium containing 10 mM sodium acetate, 1 mM MgSO₄, and 100 µM CaCl_₂_ to achieve an OD_600_ of 0.2. The aliquots were divided into treatment groups: (i) metabolites and/or antibiotics group and (ii) untreated control group (no additives), followed by incubation at 30°C with shaking at 200 rpm for 10 h. After incubation, cells were pelleted (8,000 rpm, 5 min) and resuspended in phosphate-buffered saline to a density of 10⁶ CFU/mL. SYTO-9 green fluorescent dye (Invitrogen) was added to a final concentration of 50 µM, and samples were incubated at 30°C for 15 min in the dark. The stained cells were immediately analyzed using a BD FACSCanto II flow cytometer (BD Biosciences, USA) to measure green fluorescence intensity, with all samples being directly transferred to flow cytometry analysis tubes for detection. Each experiment was repeated independently at least three times.

### Delayed resistance development

The delayed resistance development assay was conducted following established methods (34). Three colonies each of strains LTB4 and WY28 were selected from TSB agar plates and cultured in 5 mL of TSB broth at 30°C with shaking (200 rpm) for 24 h to reach stationary phase. Bacterial cells were then collected by centrifugation (8,000 × *g*, 3 min), washed three times with 0.85% saline, and resuspended in M9 minimal medium to an OD_600_ of 0.2. The bacterial suspensions were allocated to three experimental groups: (i) blank control (untreated), (ii) 1/2 MIC tobramycin, and (iii) 1/2 MIC tobramycin supplemented with 10 mM aspartate. Following 10 h of incubation at 30°C with agitation (200 rpm), cells were harvested by centrifugation, transferred to fresh antibiotic-free TSB medium, and cultured for an additional 12 h. This sequential passage protocol was repeated for 16 cycles, with MIC determinations performed after each cycle.

## Data Availability

The data of the metabolome of asparate have been uploaded to MetaboLights under membrane MTBLS12725.

## AFTER EPUB

*[This article was published on 28 August 2025 with an error in the second author's given name. The name was corrected in the current version, posted on 9 September 2025.]*

## SUPPLEMENTAL MATERIAL

Supplemental material msystems.00794-25-s0001.docx Table S1; Fig. S1 to S8.

ASM does not own the copyrights to Supplemental Material that may be linked to, or accessed through, an article. The authors have granted ASM a non-exclusive, world-wide license to publish the Supplemental Material files. Please contact the corresponding author directly for reuse.
