# Supplementary material for: Aspartate potentiates tobramycin against multidrug-resistant Edwardsiella tarda through enhancing proton motive force and membrane permeability
Source: mSystems. 2025 Aug 28;10(9):e00794-25. doi: 10.1128/msystems.00794-25 (PMC12456000; doi:10.1128/msystems.00794-25)
Supplement: Supplemental material — Table S1; Fig. S1 to S8. [file msystems.00794-25-s0001.docx]

Supplementary Table 1 qRT-PCR primers of *Edwardsiella tarda*

| **gene** | **sequence (5'-3')** | **gene** | **sequence (5'-3')** |
| --- | --- | --- | --- |
| 16S-F | GTAGTCCACGCTGTAAACGA | *aspC*-F | ACGGCTGTTGCCATAACCC |
| 16S-R | GAATTAAACCACATGCTCCA | *aspC*-R | CAAAGCCCTGATAGGCGAAGT |
| *ansA*-F | CAACCTGACCCAGTGCTTCTCC | *purB2*-F | ATCCTCCCTGACCGCCG |
| *ansA*-R | TGCCTCAACCGTCATGTCAAA | *purB2*-R | GCAGCCAACGCACTTCTACC |
| *asnB*-F | GTTTGTCGCCTCCGAGATGA | *argH-F* | *GGCGACCGATCTGAAGCTATG* |
| *asnB*-R | GCGGTGTCGCTAGGGTTGT | *argH-R* | *GCCAGACACCAATGGGCAA* |
| *asnA*-F | GCAGCGTCTCGGTCTCATTG | *pckA-F* | *GCCTCCTACGGCATTTCTGAC* |
| *asnA*-R | GCGGCAGGTTCTTCACCTTTA | *pckA-R* | *GGTTGGTGCATTTGGCTCC* |
| *ETAE_2391*-F | AACGGCGTCTCCGAGCTTAT | *ETAE_1447-F* | *CAAGGGCGAAGGCGATAA* |
| *ETAE_2391*-R | TGCTCATCGCAGCGGTAGTG | *ETAE_1447-R* | *GGGAAGGAGACGGCCAGA* |
| *lysC*-F | GGCGTCACCAACCACCTCAT | *maeB-F* | *ATCCCAAGCGGGTCGTG* |
| *lysC*-R | CGTTCAATTTCACTGCGTACCAC | *maeB-R* | *CGTAATAGGTTTGCCAATAGTCGT* |
| *sdaA*-F | GGTTCCCTATCCGTTCCACTC | *ETAE_1161-F* | *ACGAGACCCTGTTCTACCGC* |
| *sdaA*-R | ATGCCTTCGGTGTTCAGTCC | *ETAE_1161-R* | *GTGACCACGATCACCTTTACGT* |
| *sdaB*-F | CATCGGCACGCTGTATAAGATG | *aceE-F* | *TGGACGCACCACCCTGAAC* |
| *sdaB*-R | ATGCACGGCACCTGAACCT | *aceE-R* | *CGCTGCCAACGCCATACTC* |
| *ilvA*-F | CGTAAGGGCAGCTTCCTGG | *gltA-F* | *TATATTGACGGCGATGAGGG* |
| *ilvA*-R | CGTCTCATCGTGGCACTCATAG | *gltA-R* | *TATTCTGCGGGTAGATAAAGGG* |
| *tdcB*-F | CGGTGCGTTCAATAAGCTGA | *acnB-F* | *ACACGGCTGCCTATCCCAA* |
| *tdcB*-R | AATGATACGGATGGTCGGGTT | *acnB-R* | *GGCTGCATCTGCCCTTTAAA* |
| *dsdA*-F | CGCTGTTCGTCTACCTGCC | *ETAE_2050-F* | *GACGCCGATTATTCCCTTCA* |
| *dsdA*-R | GAATGCCCTCCTCCTGTGC | *ETAE_2050-R* | *GACCTGGGTCGCCTTCTCTC* |
| *ETAE_2409*-F | CATCCTTGCCTGTTCGAGTATG | *sucB-F* | *TTCTGGTTCCCGATCTGCC* |
| *ETAE_2409*-R | CGTCCCACATAGCGGTTTTT | *sucB-R* | *CGTTACCGTTGCTCCCTCC* |
| *purD*-F | GGCGAACACGTGCTACCG | *sucD-F* | *AAAGGGGGCAGCGAACAT* |
| *purD*-R | GCCAAATCACCTGCTCCATT | *sucD-R* | *CCTGGAACAGCGCCAGAA* |
| *ETAE_1034-*F | GTAGAGGGGCGTCTGGAGG | *sdhA-F* | *GACCCGTTCCCATACCGTT* |
| *ETAE_1034*-R | ATGCTGGGTCACATTGGGG | *sdhA-R* | *TGAAGTAGACCACCTCGCCG* |
| *nagD*-F | AGGAGATGCCGCTGGTGAT | *fumC-F* | *GAGCATAGCGGGCAACATATC* |
| *nagD*-R | GGGGTTGGTGGCGATAAAG | *fumC-R* | *CACCAGCGGACGAAAGACA* |
| *surE*-F | CGAAGAGACCGATTTCGCC | *mdh-F* | *TCATCCGCTCCAACACCTTC* |
| *surE*-R | CACCGCCCTTATCCACCC | *mdh-R* | *TACCAGCCCCTCTTTACCCA* |
| *ETAE_3270*-F | CGGGTACAGGTGCGTCAGG | *gltB-F* | *CATGAACGGGACAACTGCG* |
| *ETAE_3270*-R | AAAGCGGTGGGTGGAATAGA | *gltB-R* | *CGGCGGGCGATAAACA* |
| *add*-F | GAGCTGCGCTTTTCTCCCTA | *gltD-F* | *CTATCGGCAGCGTAGAGCG* |
| *add*-R | CCTGCCAGATGCTTTCCG | *gltD-R* | *GGGAATGCCAAAGGTGAGC* |
| *ETAE_0921*-F | GTGAAAGGGTTAATTGGCGTATT |  |  |
| *ETAE_0921*-R | CGGCGGTTCTGTCTGGGAT |  |  |
|  |  |  |  |


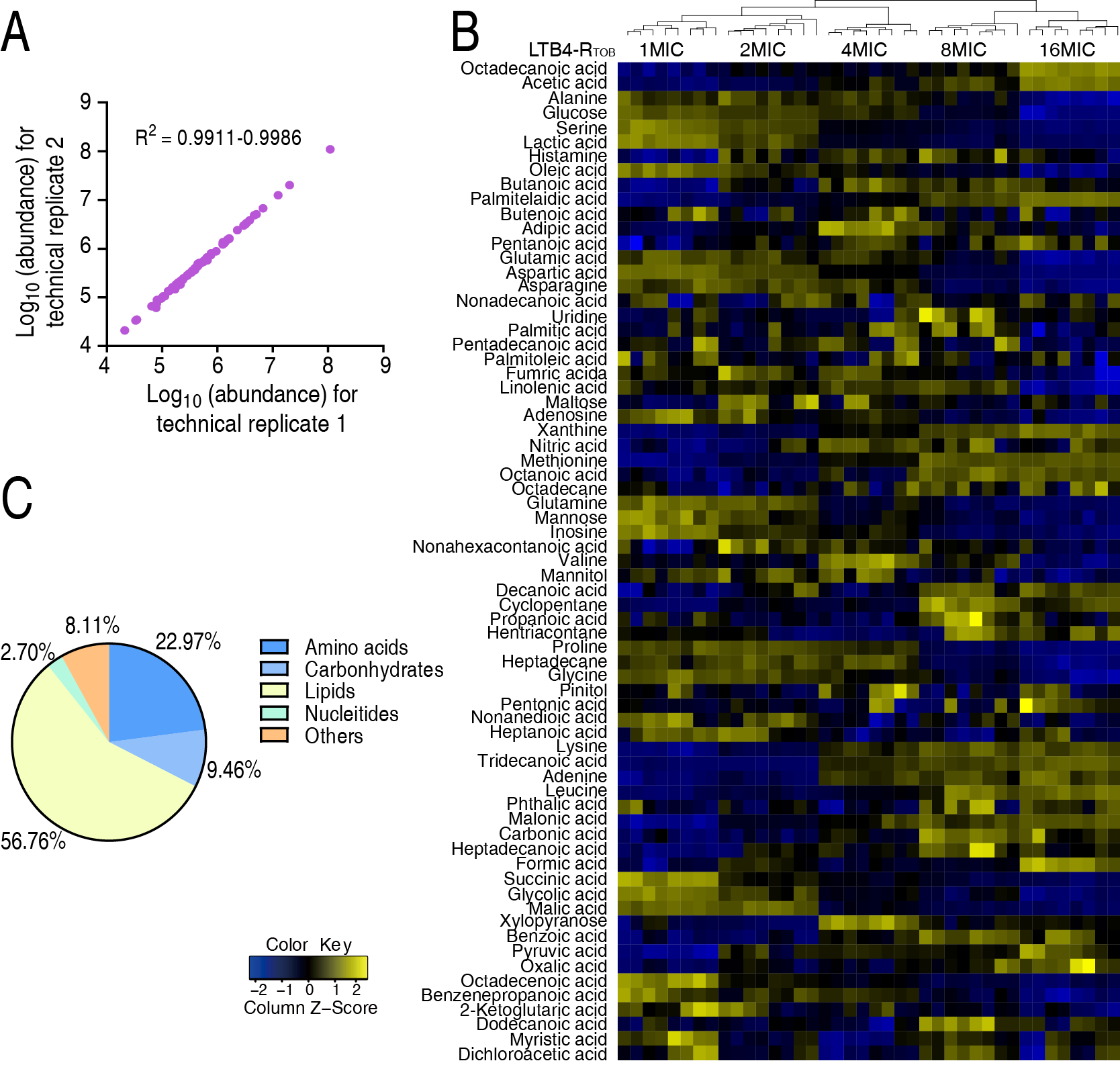


Figure S1 Dynamic metabolic profiles in LTB4-R_TOB_.

A．Correlation coefficient between technical replicates.

B. Heat map of unsupervised hierarchical clustering of all metabolites (row). Yellow and blue colors indicate increase and decrease of the metabolites scaled to mean and standard deviation of row metabolite level, respectively (see color scale).

C. Categories of all identified metabolites.


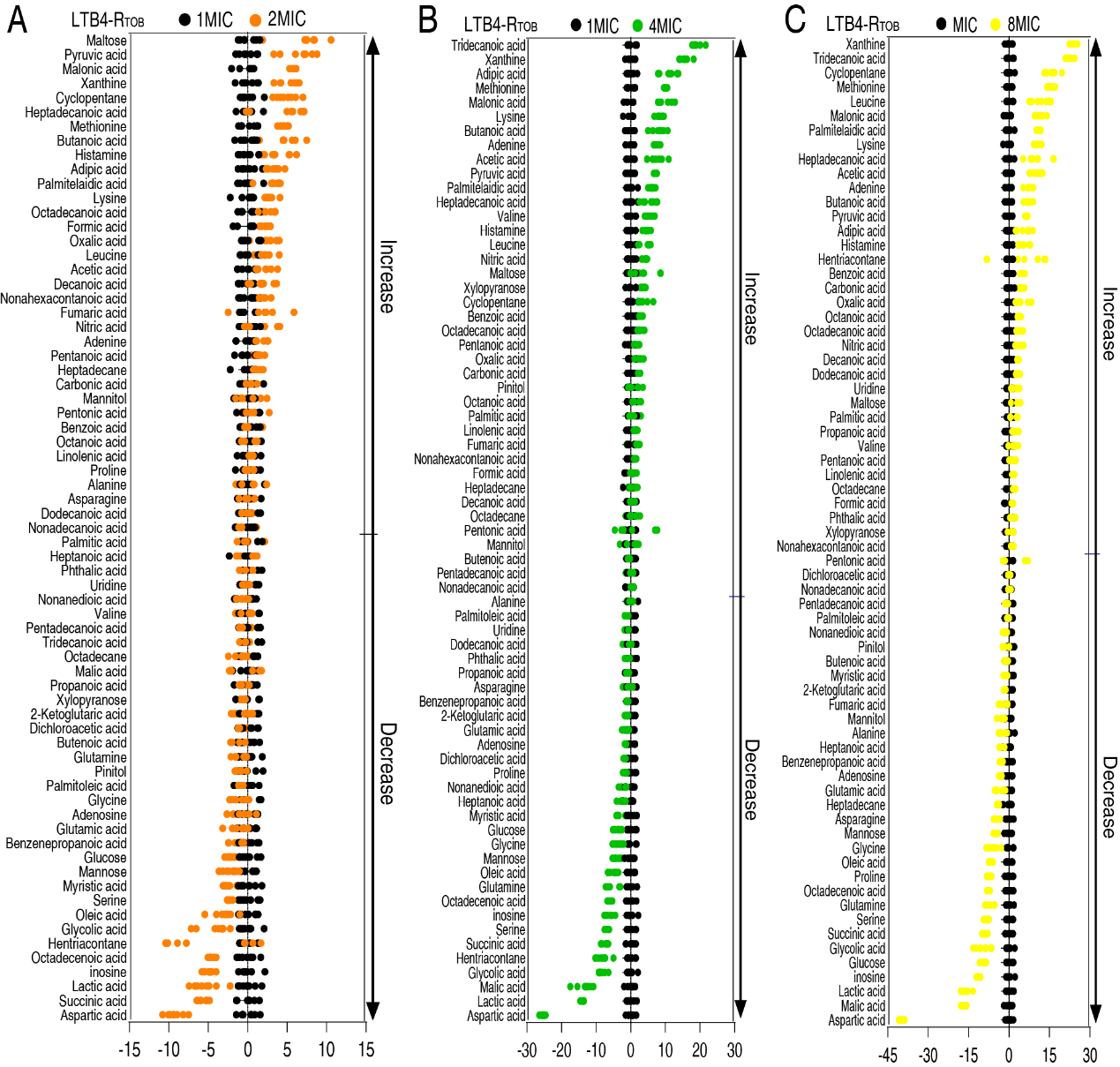


Figure S2 Z-score plot of differential metabolites in LTB4-R_TOB_ with in LTB4-R_TOB-2MIC_ (A), LTB4-R_TOB-4MIC_ (B), LTB4-R_TOB_-_8MIC_ (C) separately scaled to the mean and standard deviation of control LTB4-R_TOB-1MIC_.


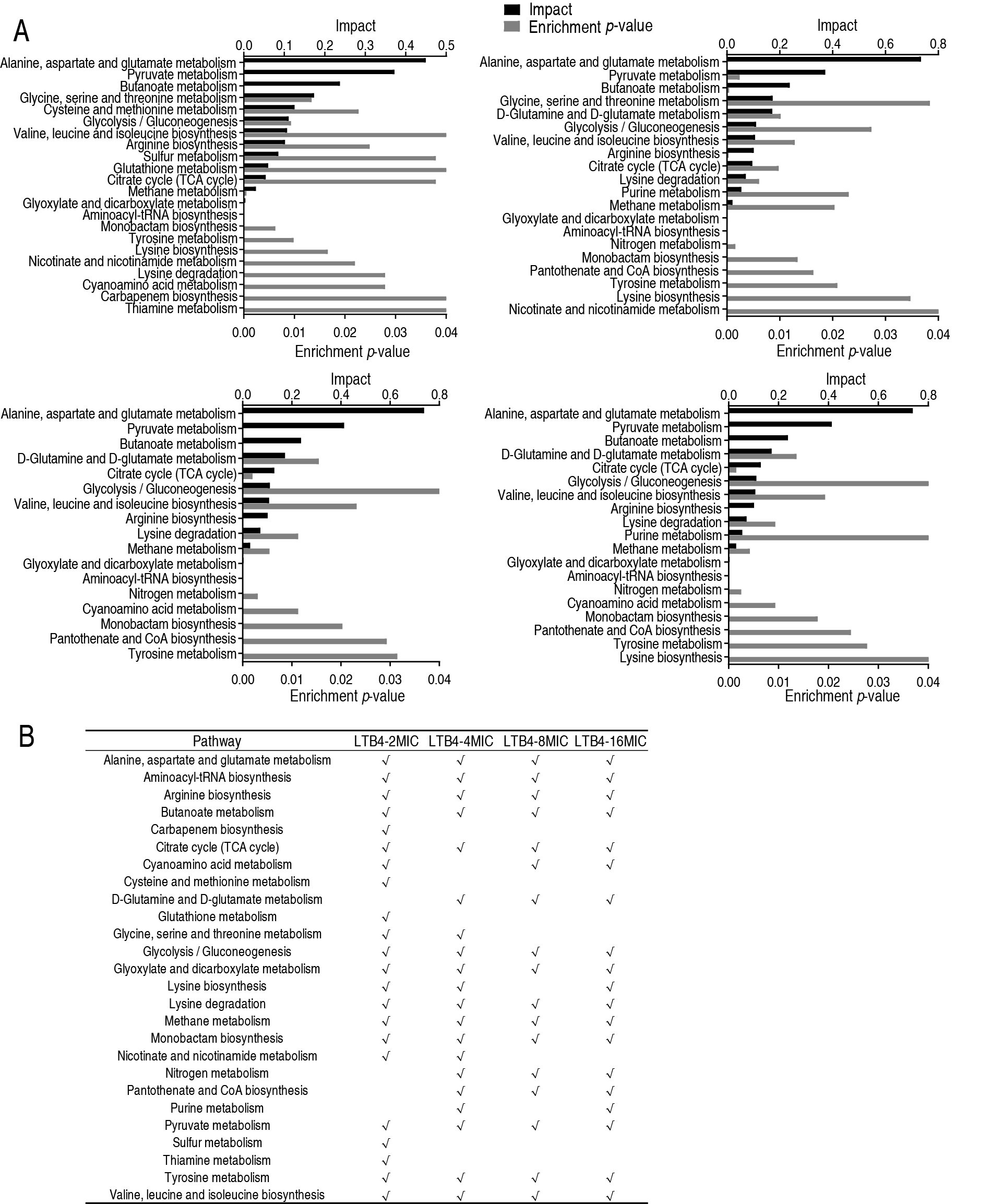


Figure S3 Pathway enrichment analysis in LTB4-R_TOB_.

A. Pathway enrichment in LTB4-R_TOB-2MIC_ (Upper left), LTB4-R_TOB-4MIC_ (Upper right), LTB4-R_TOB_-_8MIC_ (down left) and LTB4-R_TOB_-_16MIC_ (down right)_._

B. Collection of data A showing overlapped enriched pathways.


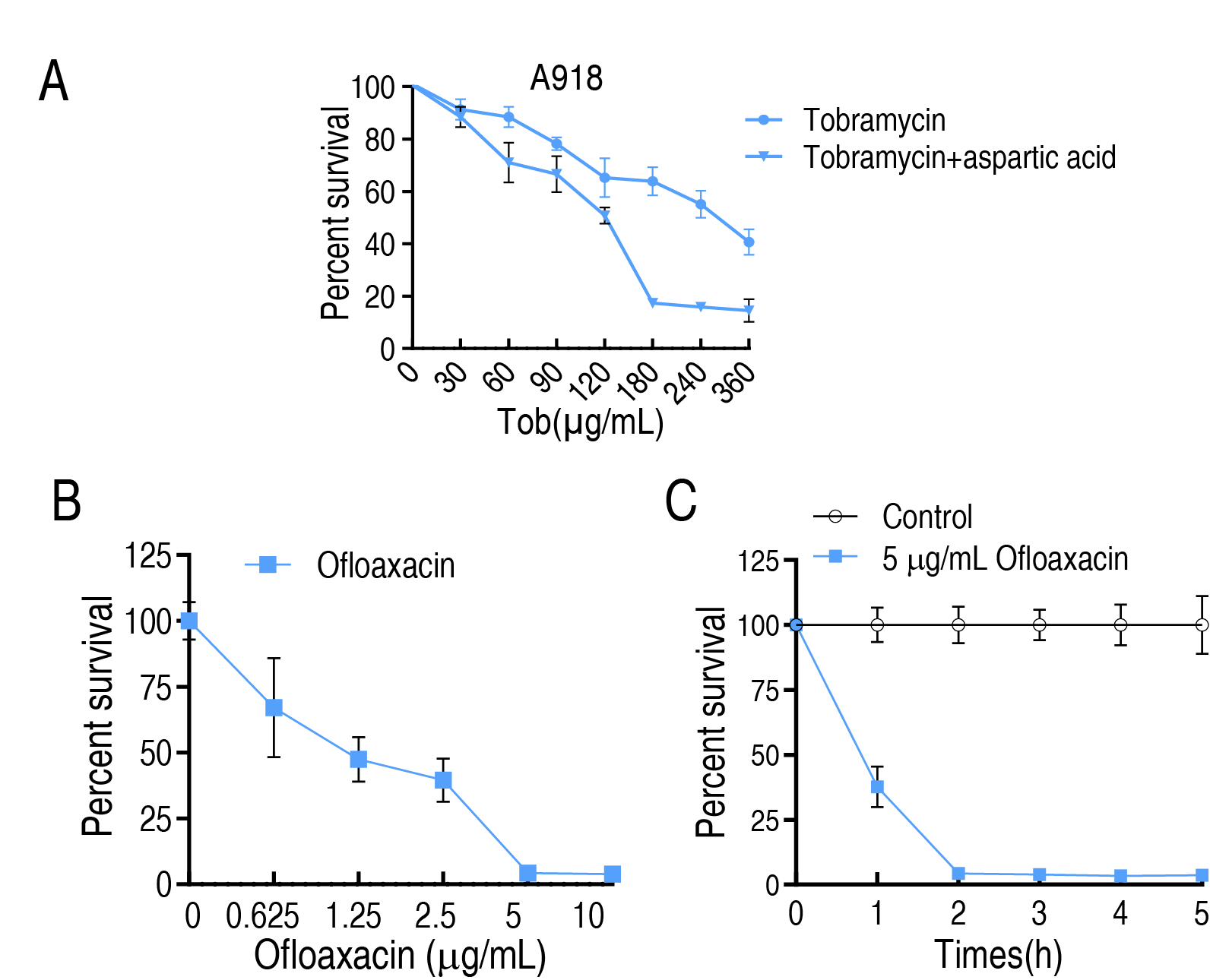


Figure S4 Percent survival of A918 in the indicated concentrations of tobramycin plus 10 mM aspartate (A) and the optimal ofloxacin concentration (B) and treatment duration (C) for preparing persisters.


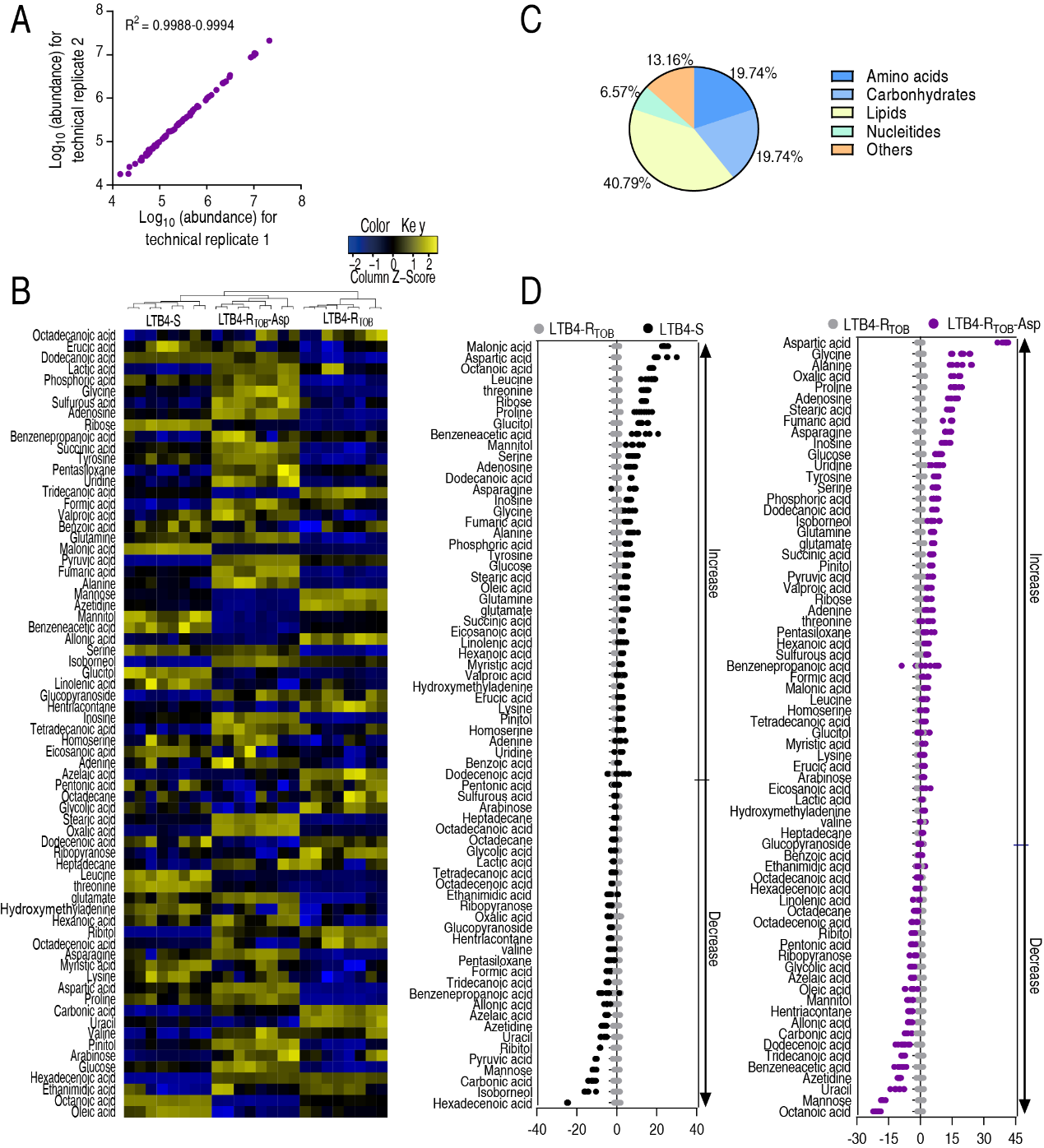


Figure S5 Aspartate reprograms metabolomes in LTB4-R_TOB-16MIC_.

A．Correlation coefficient between technical replicates.

B. Categories of all identified metabolites.

C. Heat map of unsupervised hierarchical clustering of metabolites (row). Yellow and blue colors indicate increase and decrease of the metabolites scaled to mean and standard deviation of row metabolite level, respectively (see color scale).

D. Z-score plot of differential metabolites.


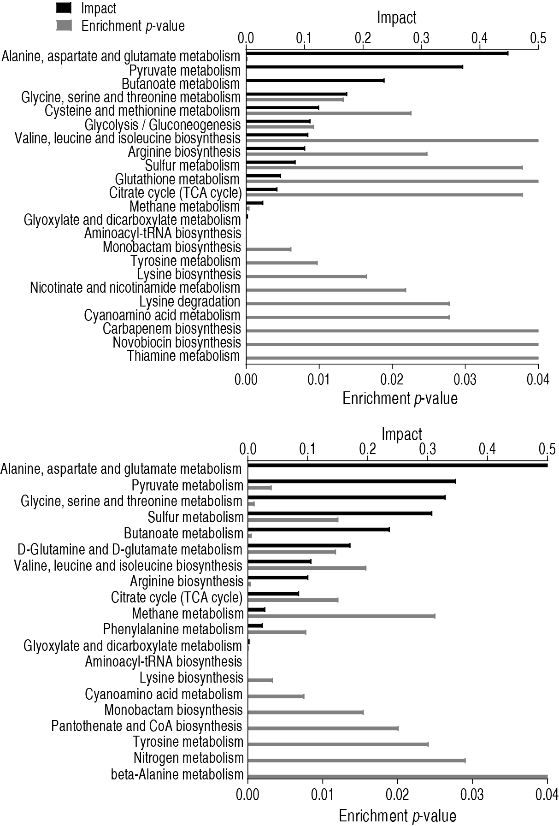


Figure S6 Pathway enrichment in LTB4-S (left) and LTB4-R_TOB-16MIC_ + Asp (right) compared to LTB4-R_TOB-16MIC_.


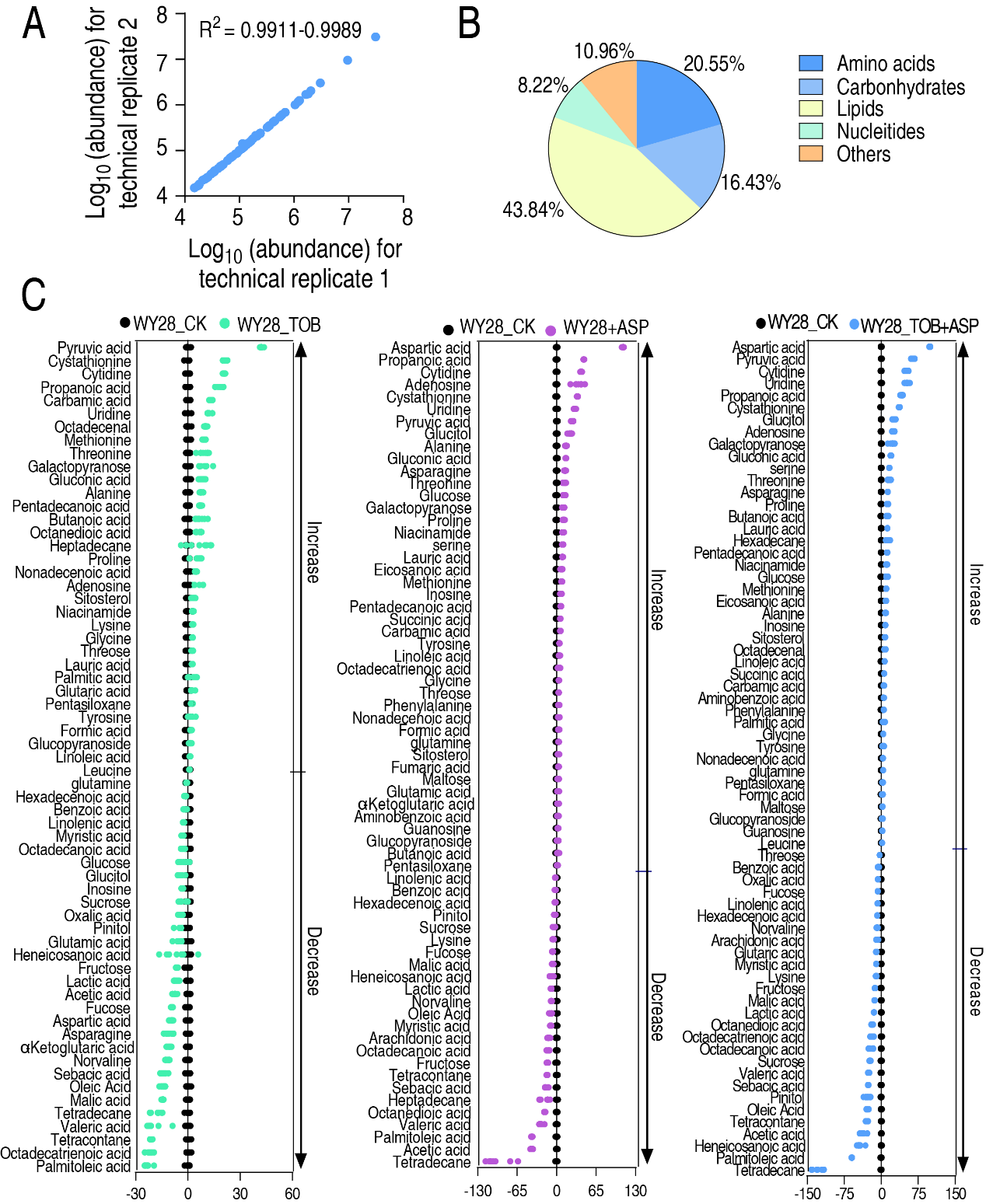


Figure S7 Aspartate reprograms metabolomes in WY 28.

A. Correlation coefficient between technical replicates.

B. Categories of all identified metabolites.

C. Z-score plot of differential metabolites.

**
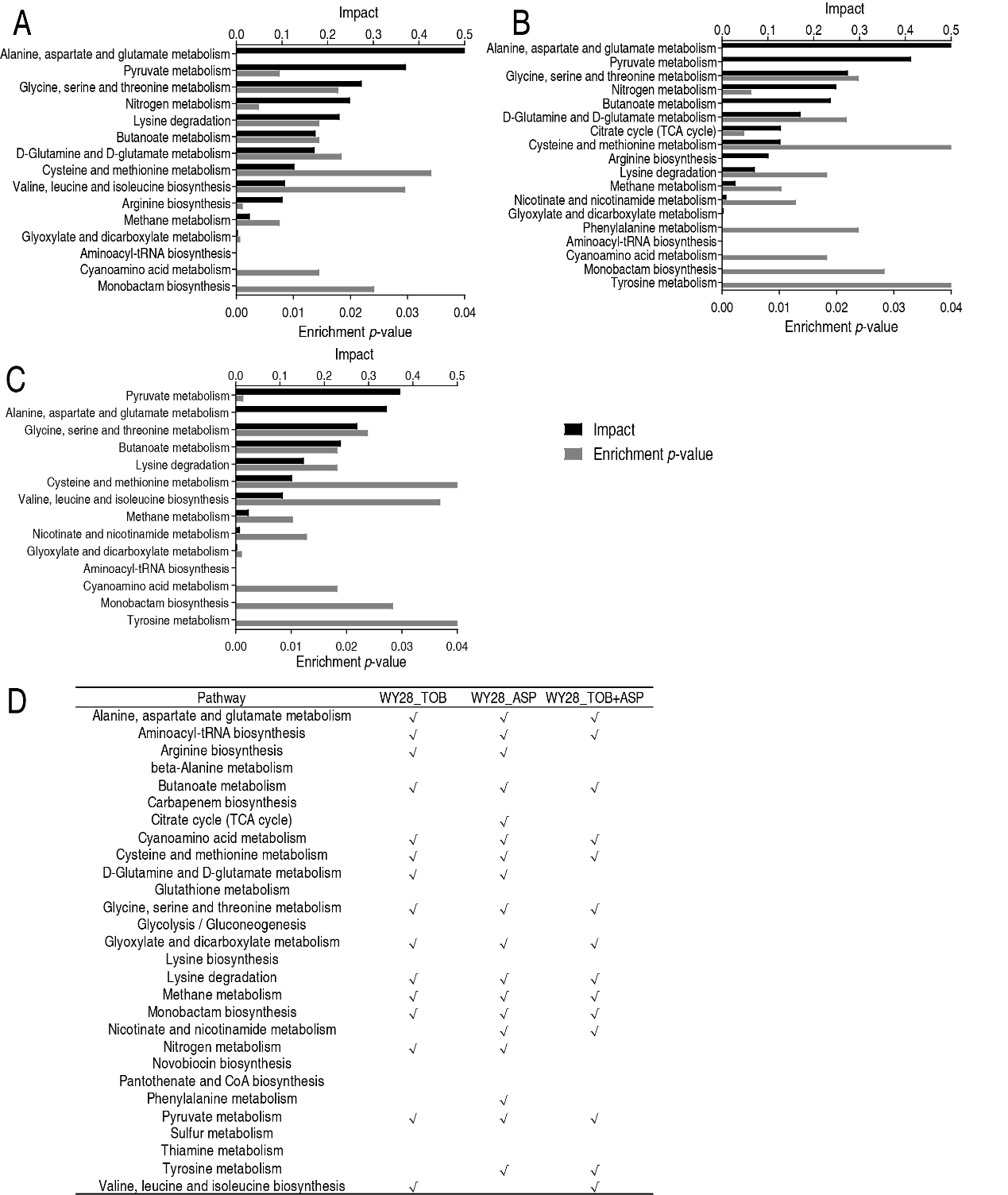
**

Figure S8 Pathway enrichment analysis in WY28.

A. Pathway enrichment in WY28 and WY28_TOB (A), WY28 and WY28 + ASP (B), and WY28 and WY28_TOB+ ASP (C)._._

D. Collection of data (A) showing overlapped enriched pathways.
